# Chiral domain wall motion in unit-cell thick perpendicularly magnetized Heusler films prepared by chemical templating

**DOI:** 10.1038/s41467-018-07091-3

**Published:** 2018-11-07

**Authors:** Panagiotis Ch. Filippou, Jaewoo Jeong, Yari Ferrante, See-Hun Yang, Teya Topuria, Mahesh G. Samant, Stuart S. P. Parkin

**Affiliations:** 1 IBM Research - Almaden, San Jose, CA 95120 USA; 2 Max Plank Institute for Microstructure Physics, Weinberg 2, 06120 Halle (Saale), Germany; 3 New Memory Technology Lab, Semiconductor R&D Center, Samsung Electronics, Milpitas, CA 95053 USA

## Abstract

Heusler alloys are a large family of compounds with complex and tunable magnetic properties, intimately connected to the atomic scale ordering of their constituent elements. We show that using a chemical templating technique of atomically ordered X′Z′ (X′ = Co; Z′ = Al, Ga, Ge, Sn) underlayers, we can achieve near bulk-like magnetic properties in tetragonally distorted Heusler films, even at room temperature. Excellent perpendicular magnetic anisotropy is found in ferrimagnetic X_3_Z (X = Mn; Z = Ge, Sn, Sb) films, just 1 or 2 unit-cells thick. Racetracks formed from these films sustain current-induced domain wall motion with velocities of more than 120 m s^−1^, at current densities up to six times lower than conventional ferromagnetic materials. We find evidence for a significant bulk chiral Dzyaloshinskii–Moriya exchange interaction, whose field strength can be systematically tuned by an order of magnitude. Our work is an important step towards practical applications of Heusler compounds for spintronic technologies.

## Abstract (web summary)

Heusler compounds are of great interest for spintronic applications. Here the authors report current driven domain wall motion in unit cell thick perpendicularly magnetized Heusler films with low current densities and show the velocity is dominated by the bulk chiral Dzyaloshinskii–Moriya exchange interaction.

## Introduction

Spintronic materials and phenomena are at the heart of several novel memory and memory-storage technologies that could allow for scaling to dimensions beyond those possible using conventional charge-based devices. However, to make this possible will require the development of magnetic materials with stringent specifications on their properties. For advanced magnetic random access memories (MRAM), magnetic materials with high perpendicular magnetic anisotropy (PMA) are needed to overcome the superparamagnetic limit. In addition, for emerging domain wall memory devices^1,2^, high PMA is also needed to obtain sufficiently narrow domain walls (DWs) to allow for high density. An attractive set of tunable, multi-functional magnetic materials is the family of Heusler compounds, which are of the form X_2_YZ and X_3_Z, where X and Y are transition metals and Z is a main group element^3,4^. A subset of these compounds stabilizes in a tetragonal phase^5,6^ which can display large PMA, but until now in their thin film form, PMA has only been observed in films too thick to be useful technologically^7–10^. Here we show that ultrathin Heusler layers, as thin as 1 unit cell, can be prepared at ambient temperature with excellent magnetic properties by using a chemical templating technique. Previously, Suzuki et al. showed that 1 nm thick layers of the L1_0_ ordered MnGa compound could be grown using a CoGa buffer material^11^ but our work far extends this earlier work to provide a universal method for the growth of atomically ordered binary and ternary compounds, including both L1_0_ and, more importantly, Heusler compounds, whose primary structure is composed of alternating atomically thick layers with different chemical characteristics. Rather than the speculation by Suzuki et al. that a Ga termination layer played a critical role in the growth of L1_0_-MnGa, we show that it is due to the chemical templating of successive atomic layers by the two distinct atomic layers in ordered CoGa. We demonstrate the universality of this chemical templating principle with the examples of CoAl, CoGe, CoGa, CoSn, and introduce the basic principle by which these chemical templating layers (CTLs) can be identified, and, thereby, make predictions about many other possibilities. We find that CoAl is by far the best of those that we have explored and is effective over a wide range of preparation temperatures, even as low as ambient temperature, whereas the other layers are only effective at much higher temperatures. We believe that the CTL growth technique will motivate the technological application of Heusler compounds, especially since we also demonstrate that the CTL technique even works for growth on amorphous substrates. We note that it is more difficult to achieve atomic ordering in compounds with metallic bonding than, for example, in compound semiconductors or complex oxides where a variety of thin film growth techniques have been used to grow beautifully ordered materials^12–15^.

Another advantage of the Heusler materials studied here is that they are ferrimagnetic with low magnetization and, consequently, exhibit very narrow DWs that we estimate are as narrow as 1 unit cell. Another family of ferrimagnetic materials, with similarly low magnetization, are the amorphous rare-earth transition-metal (RE-TM) alloys^16^ but these have low Curie temperatures, and a very strong temperature dependence of their magnetic properties due to the very different temperature-dependent magnetizations of the respective RE and TM sub-lattices^17^, in contrast to the Heusler compounds.

The most extensively studied tetragonal Heusler compounds are Mn_3_Ga and Mn_3_Ge because they display very high uniaxial magnetic anisotropy in their bulk form and very large PMA in thin films for which the *c*-axis is oriented perpendicular to the film^8,18–22^. But to obtain these properties, chemical ordering of the elements within the Heusler is essential. Thus, typically, films of these compounds have been grown using MgO (001) crystalline substrates with epitaxial seed layers of bcc Cr (001)^8,22^ or fcc Pt (001)^21^, where the Heusler films are then deposited and annealed at high temperatures in order to be chemically ordered. These methods are ineffective for layers thinner than ~5 nm due to interdiffusion between the seed and Heusler layers that damages the magnetic properties. Here we show that by using chemical templates that are formed from chemically ordered X′Z′ (X′ = Co; Z′ = Al, Ga, Ge, Sn) layers, excellent magnetic properties are obtained in Mn_3_Ge, and several other binary and ternary Heusler compounds, for layers only 1 unit cell thick, even when the Heusler layers are deposited at ambient temperature without additional annealing steps.

## Results

### Chemical templating

**Fig. 1:**
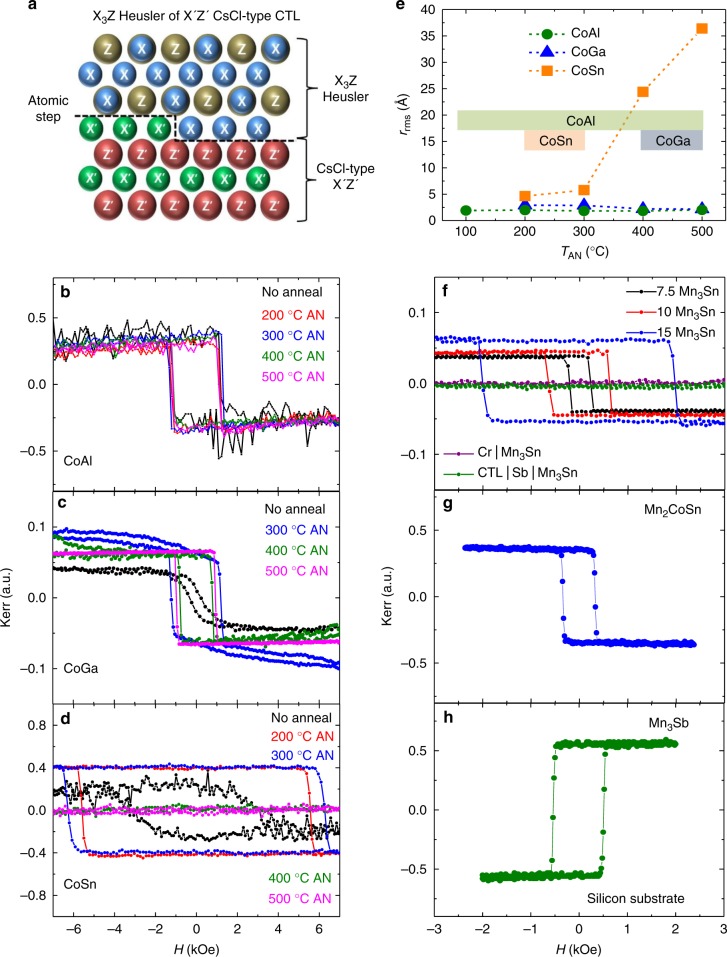
Ultrathin tetragonal Heusler films on CTLs. **a** Schematic illustration of the atomic templating concept viewed along the CTL [100] and Heusler [110]. X and X′ are transition metal elements and Z and Z′ are main group elements. **b** Dependence of CoAl CTL annealing temperature on P-MOKE hysteresis loops for 20 Å thick Mn_3_Ge Heusler films deposited on MgO (001)|20 Å MgO|300 Å CoAl. **c** Dependence of CoGa CTL annealing temperature on P-MOKE hysteresis loops for 20 Å-thick Mn_3_Ge Heusler films deposited on MgO (001)|20 Å MgO|300 Å CoGa. **d** Dependence of CoSn CTL annealing temperature on P-MOKE hysteresis loops for 20 Å-thick Mn_3_Sn Heusler films deposited on MgO (001)|20 Å MgO|400 Å Cr|300 Å CoSn. **e** Annealing temperature dependence of *r*_rms_ for CoAl, CoGa, and CoSn CTLs. The bars in the figure show the range of annealing temperatures for which the various CTLs can be used. **f** Mn_3_Sn thickness dependence of P-MOKE hysteresis loops for 7.5, 10, and 15 Å Mn_3_Sn thick layers deposited on MgO (001)|20 Å MgO|300 Å CoGa CTL. Also shown, for a 15 Å Mn_3_Sn thick layer, the P-MOKE hysteresis loops for a Cr seed layer (MgO (001)|20 Å MgO|400 Å Cr|15 Å Mn_3_Sn) and for a 2 Å Sb insertion layer (MgO (001)|20 Å MgO|300 Å CoGa CTL|2 Å Sb|15 Å Mn_3_Sn). **g** P-MOKE hysteresis loop for 20 Å-thick ternary Heusler Mn_2_CoSn film deposited on MgO (001) | 20 Å MgO|300 Å CoGa CTL. **h** P-MOKE hysteresis loop for 20 Å-thick Mn_3_Sb Heusler film deposited on Si (001)|250 Å SiO_2_|50 Å Ta|3 Å CoFeB|30 Å MgO|50 Å CoAl CTL. All films were capped with 20 Å MgO|20–30 Å Ta

The CTL concept is illustrated schematically in Fig. 1a. The underlayers are formed from non-magnetic binary alloys X′Z′ where X′ is a transition metal and Z′ is a main group element. An important prerequisite is that the in-plane lattice constant of X′Z′ closely matches that of the Heusler film (see Supplementary Table 1 for a list of potential X′Z′ candidates). Additionally, it is advantageous if X′ and Z′ are not detrimental to the magnetic properties of the X_3_Z layer in case there is some intermixing or interdiffusion. Finally, it is essential that the CTL is chemically ordered with alternating layers of X′ and Z′ so that even when there are atomic steps in the surface of this layer, the Heusler layer will automatically grow atomically ordered. As shown in Fig. 1a when the surface of the CTL is X′ terminated, then a XZ layer will preferentially grow on top of it, whereas an XX layer will grow on top of Z′ terminated regions due to the preferential chemical bonding of X′ to Z and Z′ to X. CoAl, CoGe, CoGa, and CoSn are four exemplary candidates where the chemically distinct Co and Al (Ge, Ga, Sn) atoms, form alternating atomic layers, and the lattice mismatch between CoAl, CoGe, CoGa, and CoSn with a typical tetragonal Heusler alloy is small (2.8%, −2.6%, 3.5%, and 6.7%, respectively).

CTLs formed from single crystal epitaxial films of CoAl (Co_1−*x*_Al_*x*_), CoGa (Co_1−*x*_Ga_*x*_) and CoSn (Co_1−*x*_Sn_*x*_) were prepared on MgO (001) single crystal substrates with MgO or MgO|Cr buffer layers (see Methods for growth details). CoAl, CoGa, and CoSn CTLs were deposited at ambient temperature and then, except in one case, annealed at various temperatures *T*_AN_ between 200 and 500 °C. X-ray diffraction (XRD) measurements on CTLs, reveal that these layers exhibit a B2 structure, that is comprised of alternating atomic layers of Co and Al (Ga, Sn) along the (001) out of plane direction (Supplementary Figs. 1–4). The chemical ordering of CoGa and CoSn is substantially improved with annealing but CoAl shows excellent chemical ordering even without an annealing step. Heusler compounds, in particular, Mn_3_Z layers, were deposited at ambient temperature on the CTL and capped with 20 Å MgO|20–30 Å Ta. Their magnetic properties are strongly affected by the CTL’s chemical ordering as revealed by perpendicular magneto-optical Kerr Effect (P-MOKE) measurements for Mn_3_Z grown on CTLs annealed at various temperatures. Examples are given in Fig. 1b–d, respectively, for CoAl|20 Å Mn_3_Ge, CoGa|20 Å Mn_3_Ge and CoSn|20 Å Mn_3_Sn. Excellent PMA with square hysteresis loops (remanent moment/saturation moment ~1) are found when the CTL is annealed above 400 °C for CoGa, between 200 and 300 °C for CoSn, while for CoAl, no annealing is needed although excellent PMA is also obtained for annealing between 200 and 500 °C. The properties of the CTL are strongly influenced by tiny changes in its chemical composition. Only for a narrow composition range, which for CoGa is centered about ~Co_53_Ga_47_, are the films sufficiently smooth and non-magnetic. Very smooth surfaces with a root mean square roughness, *r*_rms_, below 3 Å were found for all annealing temperatures for Co_53_Ga_47_ CTLs. Very smooth surfaces were found for films with Co_51_Sn_49_ CTLs only for *T*_AN_ ≤ 300 °C. For the films with Co_51_Al_49_, very smooth surfaces were found for all annealing temperatures (see Fig. 1e and Supplementary Fig. 5). In the following, CoGa CTL refers to the optimized composition of Co_53_Ga_47_, with a corresponding annealing temperature of *T*_AN_ = 500 °C; CoAl CTL refers to Co_51_Al_49_ with *T*_AN_ = 400 °C; and, CoSn CTL refers to a bilayer CTL composed of a first Co_53_Ga_47_ annealed at 500 °C, followed by a Co_51_Sn_49_ layer annealed at 400 °C. This bilayer is smooth even at this higher *T*_AN_.

The CTL method is very effective and allows for the growth of ultrathin Heusler layers at ambient temperature that are as little as 1 unit cell thick, and which display excellent PMA. An example is given in Fig. 1f for the case of the binary Heusler Mn_3_Sn in which magnetic hysteresis loops are shown for 1, 1.3, and 2 unit cell thick layers (7.5, 10, and 15 Å, respectively). For all cases, square hysteresis loops are found (with coercive fields that increase with thickness). The CTL technique also allows for the growth of ultrathin layers of ternary Heusler alloys with excellent magnetic properties. An exemplary case of Mn_2_CoSn is illustrated in Fig. 1g using a CoGa CTL. In prior work, the highest quality Heusler films have typically been grown using Cr seed layers on MgO substrates but, as shown in Fig. 1f, no magnetism is found in Mn_3_Sn films that are 2 unit cells thick when grown using high quality Cr underlayers (see Methods). Cr cannot promote chemical ordering of the Heusler in the way that the CTL can, even though Cr is of the same structural type (A2) and nearly of the same lattice parameter. To further test our hypothesis concerning the role of the CTL, we find that even a layer of a non-magnetic main group element (Sb) as thin as 2 Å that is inserted between the CTL and the Heusler, destroys the chemical templating effect so that no magnetism is now found for a 2 unit cell thick Mn_3_Sn layer (Fig. 1f).

Furthermore, most importantly for technological applications, we have found that the CTL technique can be used to grow ultrathin Heusler layers even on amorphous substrates. An example of the growth of a 20 Å-thick Mn_3_Sb layer using a CoAl CTL on amorphous SiO_2_ thermally grown on a Si (001) substrate is shown in Fig. 1h (see Methods).

**Fig. 2:**
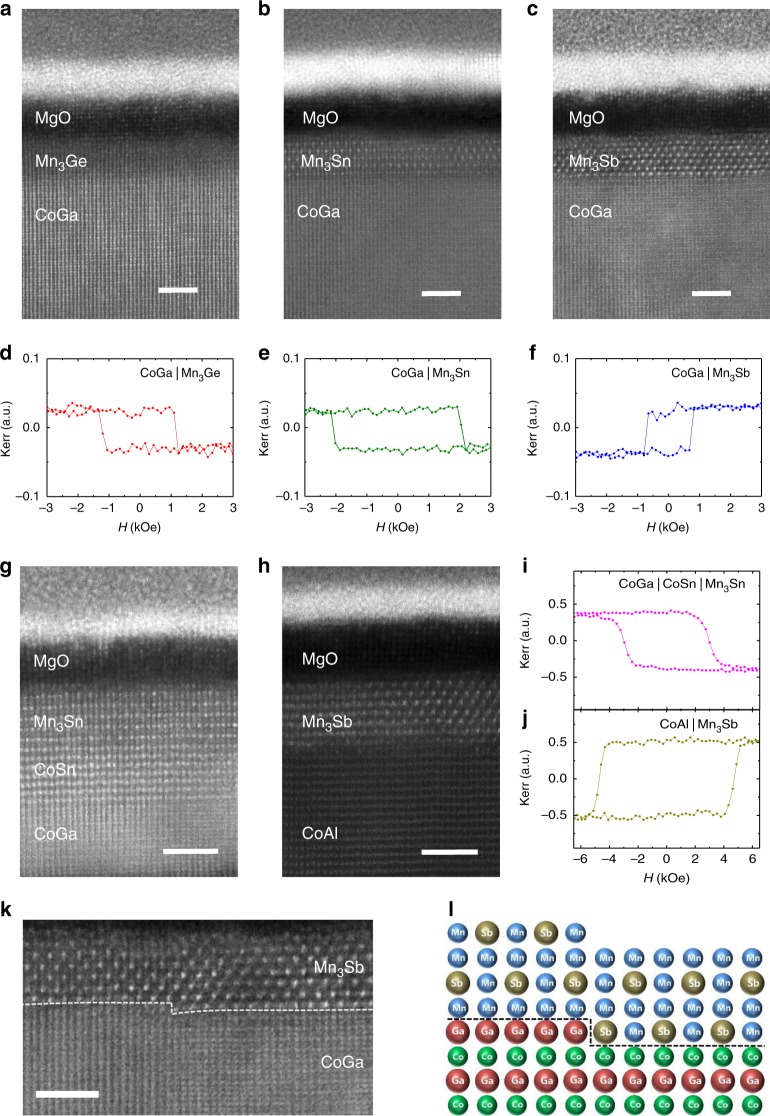
TEM images of Mn_3_Z (Z = Ge, Sn, Sb) on CoGa, CoSn, CoAl CTLs. HAADF STEM images of (**a**) Mn_3_Ge, (**b**) Mn_3_Sn, and (**c**) Mn_3_Sb Heusler films on CoGa CTL. The Heusler films were 2 nm thick and deposited at ambient temperature. Corresponding P-MOKE hysteresis loops are displayed in (**d**), (**e**), and (**f**), respectively. HAADF STEM images of (**g**) 2 nm-thick Mn_3_Sn on CoGa|CoSn CTL and (**h**) 2 nm-thick Mn_3_Sb on CoAl CTL with their corresponding P-MOKE hysteresis loops in (**i**), (**j**) respectively. An example of the growth of a 20 Å-thick Mn_3_Sb layer across a step in the CTL is shown in (**k**). The STEM image is taken along the [110] axis of the CTL (Mn_3_Sb [100]). The slight bending of the atomic planes is due to specimen charging during TEM measurements. A corresponding schematic illustration is shown in (**l**). Scale bars denote 2 nm

The chemical ordering within ultrathin Heusler layers induced by the CTL is readily observed in cross-section scanning transmission electron microscopy (STEM) images, as can be seen in Fig. 2. Excellent epitaxy of 20 Å-thick Mn_3_Z layers for three different Z = Ge, Sn, and Sb, with a CoGa CTL, is clearly seen in all cases. Moreover, for Z = Sn and Sb, the chemical ordering within the Heusler layer is obvious: the Mn–Sb and Mn–Sn layers are easily distinguishable from the Mn–Mn layers, thereby allowing the alternating atomic layers and even the chemical ordering within each Mn–Sb and Mn–Sn layer to be clearly identified. For Z = Ge, the contrast between Mn and Ge is too small, due to their similar atomic numbers, for them to be readily identified. All three samples show excellent magnetic properties as exemplified by their P-MOKE hysteresis loops (Fig. 2d–f). Similarly, the high-quality epitaxial growth of CoSn and CoAl CTLs and the associated Heusler layers is clearly shown in the STEM images in Fig. 2g, h: the corresponding P-MOKE hysteresis loops are included in Fig. 2i, j. The CTL concept is validated by the contiguity of the atomic layers within the Mn_3_Sb film across a step in the CTL that can be seen in the STEM image in Fig. 2k and the corresponding schematic image in Fig. 2l.

### Domain wall motion in ultrathin ferrimagnetic Mn_3_Z Heuslers

**Fig. 3:**
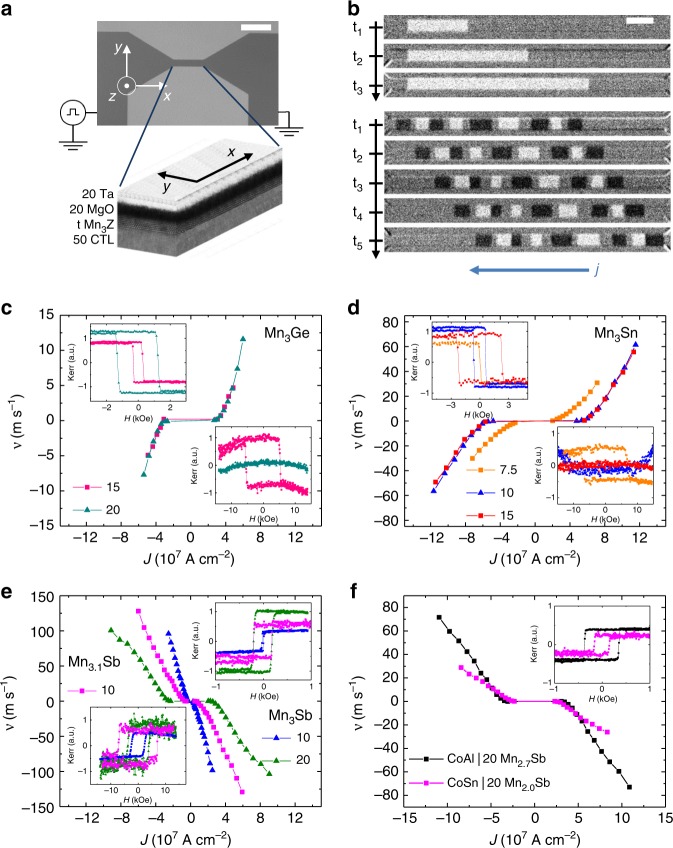
Mn_*x*_Z (Z = Ge, Sn, Sb) DW motion with CoGa, CoSn, and CoAl CTL. **a** Optical microscopy image of a nanowire device showing the contact pads at both ends (the length of the scale bar is 25 μm). A 3D structure view of the wire shows the layer stack (thicknesses are in Å). **b** Kerr images of a single DW with each frame taken after three 100 ns current pulses (top), and nine unequally spaced DWs with each frame taken after one 100 ns current pulse (lower part) for a 20 Å-thick Mn_3_Sb nanowire. The direction of current flow, *j*, is indicated by the arrow. The current density is $J = 4.7 \times 10^7\,{\mathrm{A}}\,{\mathrm{cm}}^{ - 2}$ and the length of the scale bar is 5 μm. **c**–**e** DW velocity versus current density for Mn_3_Ge, Mn_3_Sn, and Mn_3_Sb/Mn_3.1_Sb nanowires of different thicknesses with CoGa CTL. **f** DW velocity versus current density for 20 Å Mn_*x*_Sb for CoAl and CoSn CTLs. The top inset within each graph shows the corresponding out-of-plane P-MOKE loops. Bottom inset shows the in-plane P-MOKE loops

Ultrathin films of Mn_3_Ge, Mn_3_Sn, and Mn_3_Sb on CoAl, CoGa, CoSn CTLs were used to fabricate nanowires and to study the creation of DWs (see Methods) and their current-induced motion^23,24^. An optical microscopy image of a typical nanowire device is shown in Fig. 3a. The nanowire is connected to contact pads for current pulse injection. The DW motion is studied with a magneto-optical Kerr microscope (see Methods). Bright and dark contrast in the top image in Fig. 3b correspond to up ↑ and down ↓ magnetization, respectively, and grey corresponds to no change in magnetization for a 20 Å Mn_3_Sb nanowire. A series of typical Kerr images are shown at the top of Fig. 3b, after three 100 ns current pulses with current density, $J = + 4.7 \times 10^7\,{\mathrm{A}}\,{\mathrm{cm}}^{ - 2}$ are applied, three times. At the bottom in Fig. 3b, a series of 9 unequally spaced DWs are illustrated moving in the same nanowire for the same current density after a single 100 ns long current pulse is applied successively.

The dependence of DW velocity, *v*, on current density is shown in Fig. 3c–e for Mn_3_Ge, Mn_3_Sn, and Mn_3_Sb nanowires of various thicknesses on CoGa CTL and one for Mn_3.1_Sb. In each case there is a critical current density that is needed to obtain DW motion, and independent of the ↑↓ or ↓↑ DW configuration, all DWs move in the same direction and at the same speed for + and – current, but in opposite directions. The DW velocity is independent of the wire width (Supplementary Fig. 10). A most important finding is that the direction of DW motion in Mn_3_Ge and Mn_3_Sn is along the current flow whereas it is against the current flow for Mn_3_Sb. The maximum DW velocity that can be observed experimentally is limited by the maximum current that can be applied before DW nucleation takes place (subject to the maximum voltage and step size of the pulse generator). Amongst these Mn_3_Z Heuslers, the maximum velocity achieved was −103 m s^−1^ for 20 Å Mn_3_Sb and −129 m s^−1^ for 10 Å Mn_3.1_Sb. The highest DW velocities were observed for thicker Heusler films as these could sustain higher current densities before DW nucleation (~12 m s^−1^ for 20 Å Mn_3_Ge and ~60 m s^−1^ for 10 or 15 Å Mn_3_Sn) but it becomes increasingly difficult to create DWs since the coercivity increases with film thickness (see top insets in Fig. 3c–e). Figure 3f demonstrates that different CTLs can be used for the same Heusler compound: here we show DW velocity versus current density for a 20 Å-thick Mn_*x*_Sb layer using CoAl and CoSn CTLs (note that two different Mn–Sb compositions (*x* = 2.7 and 2.0) are used).

The critical current density, *J*_c_, at which the DWs start to move, is much smaller for the single-unit cell thick Mn_3_Sn and Mn_3_Sb Heusler films, due to lower intrinsic pinning, and is larger but varies little for the thicker films studied. For Mn_3_Sn, *J*_c_ is $\sim 2 \times 10^7\,{\mathrm{A}}\,{\mathrm{cm}}^{ - 2}$ for a single unit cell and $\sim 4 \times 10^7\,{\mathrm{A}}\,{\mathrm{cm}}^{ - 2}$ for thicker films. For Mn_3_Sb, *J*_c_ is very low, only $\sim 2.8 \times 10^6\,{\mathrm{A}}\,{\mathrm{cm}}^{ - 2}$, for a 10 Å film.

It is useful to compare the DW velocities that we find here with the fastest speeds reported to date in single layer magnetic materials. The fastest speeds in conventional materials are ~400 m s^−1^ in both ultrathin Co layers^23^ and Co/Ni/Co trilayer heterostructures^24^, whereas the maximum speeds we have measured in Heusler films are lower. However, this is because the maximum current that can be applied in the Heusler nanowires here is limited by their much higher resistivity. For example, if we compare the current density needed to achieve the same DW velocity (100 m s^−1^) in Co/Ni/Co layers as in our Heusler films, we find that the best result for Co/Ni/Co is a current density of $\sim 1.5 \times 10^8\,{\mathrm{A}}\,{\mathrm{cm}}^{ - 2}$^24^ versus $\sim 2.5 \times 10^7\,{\mathrm{A}}\,{\mathrm{cm}}^{ - 2}$ here (see Fig. 3e). Thus, a comparison shows that the current-induced DW motion in our ultrathin Heusler layers is ~six times more efficient than in “conventional materials”. The difference here is partly due to the ~three times lower magnetization of the Heusler compound as compared to Co/Ni/Co. But, even taking this into account, the very different mechanism for moving DWs in Heusler racetracks, where both spin–orbit torque (SOT) and spin transfer torque (STT) are comparable and together drive the DW faster, is two times more efficient than the chiral spin torque in Co/Ni/Co layers, where the STT is smaller and of opposite sign to the SOT.

The main mechanism by which the DWs are moved with current is volume spin-transfer torque which can account for the different direction of motion in Mn_3_Ge and Mn_3_Sn, as compared to Mn_3_Sb, since the volume spin polarization is calculated to be negative for the former and positive for the latter^25^. However, we find, that there are important contributions from chiral SOTs^24,26^ and that there is a significant volume (bulk) Dzyaloshinskii–Moriya exchange interaction (DMI)^27,28^ whose sign changes for the different Heuslers and sets the chirality of the DWs.

### Chiral domain wall motion mechanism

**Fig. 4:**
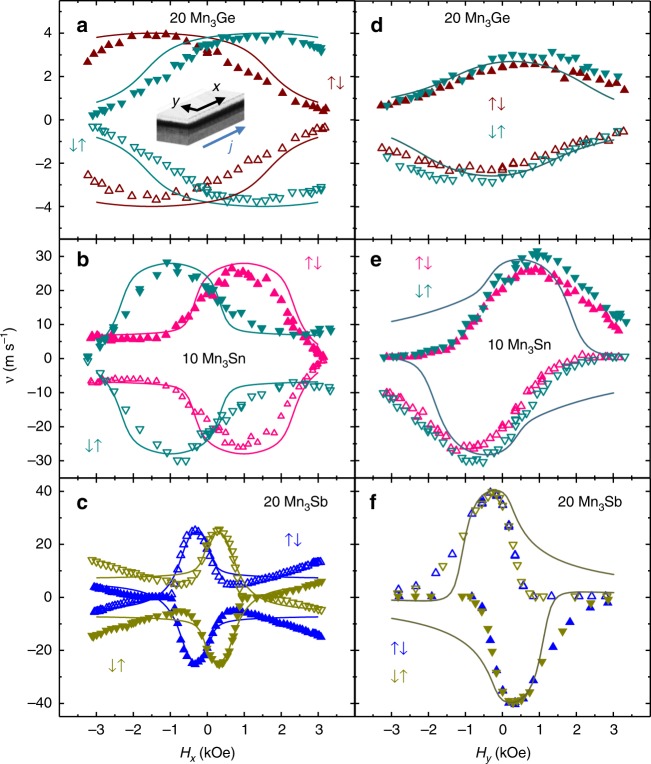
DW velocity dependence on *H*_*x*_ and *H*_*y*_. **a**–**c** ↑↓ and ↓↑ DW velocity dependence on longitudinally applied in-plane field, *H*_*x*_, for 20 Å Mn_3_Ge, 10 Å Mn_3_Sn, 20 Å Mn_3_Sb nanowires with CoGa CTL. **d**–**f** ↑↓ and ↓↑ DW velocity dependence on laterally applied in-plane field, *H*_*y*_, for the same nanowires. Positive *H*_*x*_ and *j* are along the + *x* direction, positive *H*_*y*_ is along +*y* as shown in the schematic included in **a**. Closed and open symbols represent positive and negative applied currents, respectively. Solid lines represent the 1D model simulation. Note that for the *H*_*y*_ field dependence, the simulated values for ↑↓ and ↓↑ DW configurations overlap. The current densities, *J*, used for these measurements are: (**a**) $4.7 \times 10^7$, (**b**) $8.5 \times 10^7$, (**c**) $3.1 \times 10^7$, (**d**) $4.3 \times 10^7$, (**e**) $8.6 \times 10^7$, and (**f**) $3.9 \times 10^7$, all in A cm^−2^

The SOTs are manifested especially by exploring the dependence of the DW velocity on in plane magnetic fields along (*H*_*x*_) and transverse (*H*_*y*_) to the nanowire length. Results are shown in Fig. 4a–c for *H*_*x*_ and in Fig. 4d–f for *H*_*y*_ for the ↑↓ and ↓↑ DW configurations for 20 Å Mn_3_Ge, 10 Å Mn_3_Sn, and 20 Å Mn_3_Sb on CoGa CTL. The dependence on *H*_*y*_ is independent of the DW configuration whereas the dependence on *H*_*x*_ is a mirror image for the two DW configurations. These results, reflecting the chiral DW motion, are evidence for a DMI field *H*_DM_ along the *x* direction which sets Néel type DWs. As *J* is increased from zero, the volume spin torque causes the moments in the domain wall to rotate out of the plane of the nanowire. This gives rise to a damping torque (*ɑ***M** × d**M**/d*t*) along *y* that results in a precessional motion of the DW along the nanowire. At the same time the combination of the DMI and *H*_*x*_ applied field gives rise to a torque that counteracts the volume spin torque (see Supplementary Fig. 21). The DW velocity *v* is maximum when $H_x = - H_{{\mathrm{DM}}}$ and the DW velocity decreases for increasing or decreasing in-plane fields away from this point. Thus, this accounts for the dome shaped feature in the *v* versus *H*_*x*_ curve that can be seen for all the different Heusler nanowires in Fig. 4a–c and the dome-peak position defines the magnitude (but of opposite sign) DMI field. The DW velocity goes to zero when the volume spin torque is exactly counterbalanced by the combined $H_{{\mathrm{DM}}} + H_x$ torque. Finally, there is also a contribution from a chiral SOT that arises from a spin Hall effect (SHE) in the CoGa CTL that accounts for the asymmetry of the dome on *H*_*x*_ field dependence. This SOT also accounts for the observation that the dependence of DW velocity on *H*_*y*_ is not centered about *H*_*y*_ = 0 and that the off-centering is opposite for positive and negative currents. The chiral SOT with the DMI can either add favorably (in the case of Mn_3_Sn and Mn_3_Sb) or against (Mn_3_Ge) the volume STT. Using the spin-transfer, DMI, applied field and SOTs a one-dimensional analytical model can well account for the detailed experimental results, as shown in Fig. 4 (fits to the data are shown as solid lines). In addition, the DW width is estimated to be very small, of the order of just one unit cell. The strength of the DMI in this model is estimated to be several orders of magnitude smaller than the Heisenberg ferromagnetic exchange (both referred to the net magnetization of the two antiparallel aligned ferromagnetic sub-lattices). A detailed discussion of the analytical model and the chiral-DW motion is given in the Supplementary Notes 15 and 16.

### Tailoring the SOTs and bulk DMI

**Fig. 5:**
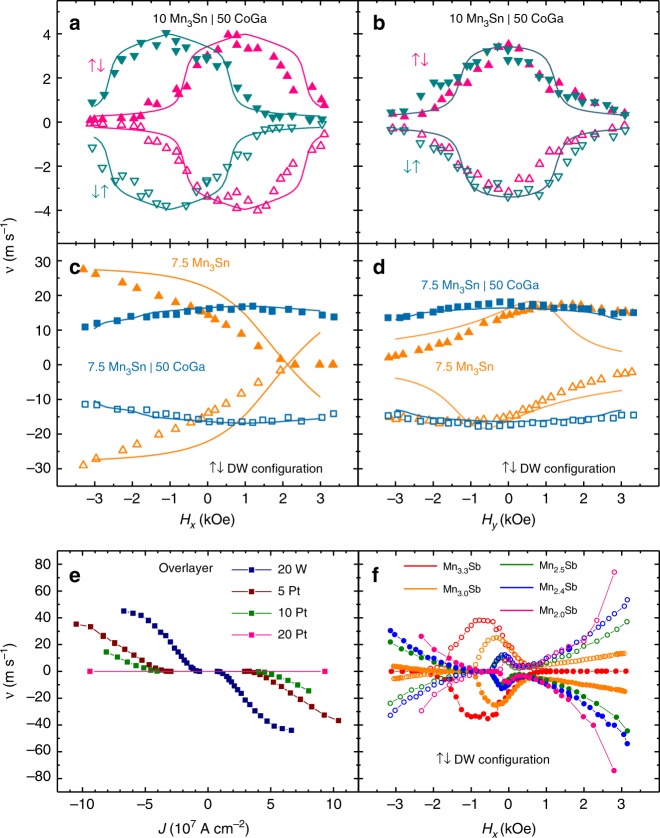
Spin–orbit torque suppression or enhancement and DMI tailoring. ↑↓ and ↓↑ DW velocity versus in-plane field *H*_*x*_ (**a**) and *H*_*y*_ (**b**) for a 10 Å Mn_3_Sn Heusler film on CoGa CTL with a 50 Å CoGa overlayer (Fig. 4b and e show corresponding data without a CoGa overlayer). In the *H*_*y*_ field dependence, the simulated values for ↑↓ and ↓↑ DW configurations overlap. The film stack is: MgO (001)|20 Å MgO|50 Å CoGa|10 Å Mn_3_Sn|50 Å CoGa|20 Å MgO|20 Å Ta and *J* used for both *H*_*x*_ and *H*_*y*_ was ~6.8×10^7^Acm^−2^. (**c**), (**d**) ↑↓ DW velocity versus in-plane fields, *H*_*x*_ and *H*_*y*_, respectively, for 7.5 Å Mn_3_Sn on CoGa CTL, with and without a CoGa overlayer. In the case of no CoGa overlayer, the stack is: MgO (001)|20 Å MgO|50 Å CoGa|7.5 Å Mn_3_Sn|20 Å MgO|20 Å Ta and *J* used for both *H*_*x*_ and *H*_*y*_ was $\sim 5.0 \times 10^7\,{\mathrm{A}}\,{\mathrm{cm}}^{ - 2}$. With a CoGa overlayer the stack is: MgO (001)|20 Å MgO|50 Å CoGa|7.5 Å Mn_3_Sn|50 Å CoGa|20 Å MgO|20 Å Ta and *J* used for both *H*_*x*_ and *H*_*y*_ was $\sim 5.7 \times 10^7\,{\mathrm{A}}\,{\mathrm{cm}}^{ - 2}$. Solid lines represent the 1D model simulation. **e** Overlayer dependence of the DW velocity versus current density for a 20 Å Mn_3_Sb film on a CoGa CTL. The stack is: MgO (001)|20 Å MgO|50 Å CoGa|20 Å Mn_3_Sb|5 Å CoGa|5–20 Å Pt or 20 Å W|20 Å MgO|20 Å Ta. **f** Mn_*x*_Sb composition dependence on ↑↓ DW velocity versus in-plane *H*_*x*_ field for the stack: MgO (001)|20 Å MgO|50 Å CoGa|20 Å Mn_*x*_Sb|20 Å MgO|20 Å Ta, with *x* = 3.3, 3.0, 2.5, 2.4, 2.0 and corresponding current densities of $J = \sim 5.9,3.1,3.1,2.9,2.8 \times 10^7\,{\mathrm{A}}\,{\mathrm{cm}}^{ - 2}$. In all cases, closed and open symbols represent positive and negative applied currents, respectively

An important point is whether the origin of the DMI exchange field is interfacial^29^ or bulk. Since an interfacial DMI is innately chiral^24^, by introducing a capping layer that is identical to the CTL, these two interfaces should compensate one another, thereby eliminating any interfacial DMI. As shown in Fig. 5a, the dependence of the DW velocity on *H*_*x*_ and *H*_*y*_ for a 10 Å Mn_3_Sn nanowire with such a capping layer has a similar dome-like shape to that shown above in Fig. 4b for the same structure without the capping layer. The center of the dome is unchanged, thereby demonstrating that the DMI is predominantly bulk in origin. However, we do find distinct differences, with and without the capping layer, that arise from the suppression of the SOT. In particular, comparing Fig. 4e with Fig. 5b (with the capping layer), the dome is no longer asymmetric and the dependence on *H*_*y*_ is clearly centered around *H*_*y*_ = 0. Also, the dependence on the current direction is eliminated, which very nicely illustrates that the contribution from the chiral spin torque disappears due to a compensation of the spin Hall effect from the second interface with the CoGa capping layer.

To further explore the role of the interfacial SOT we study a thinner nanowire composed of a single unit cell (7.5 Å) of Mn_3_Sn with and without an identical to the CTL capping layer, in which, the respective contribution of the chiral spin torque will be comparatively larger, with respect to case of the thicker 10 Å Mn_3_Sn. Results for the ↑↓ DW configuration are shown in Fig. 5c, d and for both ↑↓, ↓↑ DW configurations in Supplementary Fig. 12. Indeed, without a capping layer (in Fig. 5c, orange symbols), we can see that the dome is now obscured by the SOT that increases monotonically with *H*_*x*_ for the nanowire. When a capping layer that is identical to the CTL is introduced, the dome now becomes visible as the SOT is again eliminated (in Fig. 5c, cyan symbols). Similarly, the DW velocity dependence on *H*_*y*_ shows very similar changes to those discussed above for the thicker Mn_3_Sn nanowire (Fig. 5d).

Hence, it is clear from these data that the spin Hall torque originates from the interfaces with the CoGa layers but that the DMI torque originates predominantly from the interior of the Heusler layer. We note that non-collinear magnetic structures have recently been observed in two Heusler compounds^30,31^ that suggest a bulk DMI.

The ferrimagnetic structure of the Mn_3_Z Heuslers accounts for its low magnetization values and, consequently, narrow DWs (which we estimate to be of the order of a unit cell as shown in Supplementary Tables 2 and 3) which, thereby, leads to large DMI fields even when the DMI exchange energy is small. Similarly, the chiral SOTs give rise to velocities that increase inversely with magnetization and, therefore, even a small spin Hall angle (SHA) can have a large impact. Indeed, we find that the SOTs can be enhanced or suppressed by introducing overlayers that show a high SHA, for example, Pt^32,33^ or W^34,35^. We consider the case of a 20 Å Mn_3_Sb Heusler layer. A thin CoGa capping layer of only 5 Å is introduced, so that the top interface of the Mn_3_Sb layer is not affected by the 5, 10, and 20 Å Pt or a 20 Å W layer, that was subsequently grown. The DW velocity is strongly influenced by these capping layers, as shown in Fig. 5e, with opposite effects for Pt and W, that is consistent with the opposite sign of their respective SHAs. Figure 5e shows that the Pt layer results in a reduced DW velocity with a larger reduction the larger is the Pt thickness because Pt has the same sign of the SHA as does CoGa. By contrast, the DW velocity is higher for the W overlayer, for otherwise the same structure, which has an opposite sign of SHA to CoGa. These effects are reflected more dramatically in the longitudinal field dependence of the DW velocity, as can be seen by comparing Fig. 4c with Supplementary Fig. 15a. An example of the high tunability the Heusler film structures we studied here, demonstrate, is that their bulk-DMI field can be systematically tuned by up to an order of magnitude, by varying the composition, as seen from the shift of the dome center in Fig. 5f (in the case of 20 Å Mn_*x*_Sb, see also Supplementary Tables 2 and 3).

### Conclusions

We have introduced a universal concept of a CTL that enables the growth of complex Heusler compounds that demonstrate near bulk-like properties for films as thin as a single unit cell. The CTL can be chosen from a wide range of binary compounds that are formed from a transition metal and a main group element that are chemically ordered layer by layer. We have identified CoAl as the best CTL candidate to date, which works even when prepared at room temperature on amorphous substrates. The possibility of preparing ultrathin Heusler films with PMA makes possible their technological application for a wide-range of spintronic devices. One of these devices is the Racetrack Memory^2^ whose basic principle is the current-induced motion of a series of DWs. We have shown the realization of this phenomenon in ultrathin Heusler films and have also demonstrated the important role of the DMI, the spin Hall effect, and spin-polarization of the current. What makes Heuslers so exciting is their extraordinary tunability, perhaps unrivaled by any other class of magnetic materials. They demonstrate a vast range of properties from magnetic to topological in the bulk with properties that are potentially highly useful for spintronic devices now that we have demonstrated these compounds can be prepared in ultrathin layers that are critical for some of the most interesting spintronic applications.

## Methods

### CTL and Heusler film deposition and characterization

Si (001) covered with ~250 Å of SiO_2_ and MgO(001) substrates were used for the growth of the films in this study. The MgO substrates were cleaned in an ultrasonic bath of methanol for 15 min, followed by a treatment in an isopropyl alcohol (IPA) vapor degreaser for 2 min, dried with N_2_ gas at 65 °C for 15 min, then transferred into the deposition chamber with a base pressure of ~ 2 × 10^–9^ Torr, and therein annealed at 650 °C for 30 min in ultra-high vacuum (UHV). First a 20 Å-thick MgO buffer layer was deposited at ambient temperature with ion beam sputter deposition (IBD) using 1.5 sccm of Kr. When a Cr underlayer was used, a 400 Å-thick Cr buffer layer was then grown by IBD at ambient temperature, and subsequently annealed at 400 °C for 30 min. Then a CoAl, CoGa, CoGe, or CoSn CTL was grown by dc-magnetron sputtering at ambient temperature and annealed at *T*_AN_ for 30 min. Calibration films of 300 Ta|~300 X ^’^Z^’^ (X′ = Co; Z′ = Al, Ga, Ge, Sn)|200 Ta (thicknesses in Å), deposited on Si (001) substrates covered with 250 Å-thick thermally grown SiO_2_ were used to determine the composition of the X′Z′ CTLs using Rutherford backscattering spectrometry (RBS). Vibrating sample magnetometry (VSM) on the CTLs grown on Si (001) substrates, verified that they are non-magnetic. The Mn_3_Ge films were grown by IBD whilst the Mn_3_Sn, Mn_3_Sb, and Mn_2_CoSn films were grown by dc-magnetron sputtering at ambient temperature. The 2 Å Sb insertion layer was grown by dc-magnetron sputtering. The film stack for the Heusler film for the Si substrate is: Si (001)|250 SiO_2_|50 Ta|3 CoFeB|30 MgO|50 CoAl|20 Mn_3_Sb| 20 MgO|20 Ta (all thicknesses in Å), where the CoFeB, CoAl, and Mn_3_Sb layers were grown by dc-magnetron sputtering, the MgO layers by rf-magnetron sputtering, and the Ta layers by IBD. The CoGa overlayers were grown by dc-magnetron sputtering at ambient temperature and were not subsequently annealed. The Pt, and W overlayers were also grown by dc-magnetron sputtering. Capping layers of 20 Å MgO and 20–30 Å Ta were deposited using rf-magnetron and IBD, respectively. Here ambient temperature refers to temperatures at or below 100 °C. In Supplementary Fig. 19 we show that the P-MOKE hysteresis loops of Heusler films grown at room temperature and 100 °C are nearly identical.

XRD measurements were performed using a Bruker general area detector diffraction system (GADDS) at room temperature. Atomic force microscopy measurements were carried out using a Bruker Icon Dimension with ScanAsyst system.

High-resolution XRD measurements used in Supplementary Fig. 2 were performed using a Bruker D8 Discover system at room temperature.

The high angle annular dark field (HAADF) cross-sectional scanning transmission microscopy (STEM) imaging and electron energy loss spectroscopy studies (EELS) were performed using a JEOL JEM ARM-200F STEM Cs-corrected cold FEG atomic resolution analytical microscope with a Gatan Imaging Filter Quantum post-column energy filter and a JEOL Centurio Silicon Drift Detector Energy Dispersive Spectrometer. Conventional TEM specimen preparation using mechanical sectioning, waterless grinding, dimpling, and Argon ion milling was used for Fig. 2a–c and h. TEM-ready specimens were carbon coated (1 nm) to reduce charging/beam damage for Fig. 2a–c. The TEM sample specimen for Fig. 2g was prepared using focused ion beam (FIB) milling.

A P-MOKE system was used to probe the out-of-plane component of the magnetization of the Heusler films at room temperature using an external magnetic field that was oriented either perpendicular or parallel (within ±5°) to the film plane. This ±5° deviation from the parallel to the film orientation causes the hysteretic loops observed in the in-plane configuration, as shown in the lower insets of Fig. 3c–e.

### Nanowire fabrication and Kerr microscopy

Devices were patterned from the Heusler films using standard photolithography techniques. First, a monolayer of bistrimethylsilylamine (HMDS), an adhesion promoter, was applied to the film using a Y.E.S. vacuum oven. Then, using spin coating and baking steps, a bilayer resist of ~50 nm of polydimethylglutarimide (PMGI) and ~0.62 μm SPR 670 was applied. The bilayer resist improves the lift-off process and reduces fencing effects. Argon ion milling was used to define the devices and an AlO_*x*_ refill was used to encapsulate them. The nanowires were made wider at each end to create pads that were used for electrical connections.

The DW motion is studied by first creating a DW in the nanowire and then applying a series of current pulses of variable length *t*_p_ from 2 to 100 ns and imaging the position of the DW with a magneto-optical Kerr microscope after each current pulse or pulse train. The images shown are obtained by subtracting the initial image at *t*_0_ from the image at *t*_n_ (see Methods and Supplementary Fig. 9). Kerr microscopy images were taken in differential mode, where the initial image is subtracted from each subsequent image. Thus, any changes to the initial magnetic state of the nanowire appear in these images as bright or dark regions (Supplementary Fig. 9), whereas gray regions correspond to no change. The optimum Kerr signal to noise was obtained using high contrast and exposure due to the low moment of the ultrathin ferrimagnetic Heusler films and the transparency of the MgO substrate. Averaging of the images was used to reduce noise.

DWs were injected into the nanowires using the following procedure. Initially the magnetization of the wires was saturated in an out-of-the-film-plane field of 1–3 kOe and the Kerr contrast (dark or bright) corresponding to the magnetization direction (↑ or ↓) was determined. A combination of an opposing out of plane magnetic field, much less than the coercive field, together with high current density pulses was then used to create one or more domains within the wire. The magnetic field was then increased so as to consolidate these domains into a single domain within the wire. Finally, a sequence of field and current pulses was applied to expand this domain preferentially to one side of the wire until there was only a single DW in the wire.

Nanowires with widths of 2, 5, 10, 20 μm and lengths of 25 or 50 μm were studied. A higher current density could be accessed in the shorter wires due to the limited voltage output of the pulse generator. Electrical connections were made to the contact pads by ultrasonic wire bonding using 25 μm diameter aluminum wires. Nanosecond long voltage pulses were generated by a 10,300B Picosecond Pulse Laboratory generator. Small dc currents from 0.1 to 10 μA were used to simultaneously measure the device resistance using a bias tee. For samples with very low *J*_c_, small dc currents were used (0.1–1 μA) so as to avoid moving the DWs. The dc current was used to check that the wire resistance was unchanged and that the wire was not damaged by application of the voltage pulses. The current pulse density was calculated from the pulse voltage (multiplied by 2), nanowire resistance and width and thickness of the conducting layers (CTL, Heusler and overlayers CoGa, Pt, W). The 20 Å Ta capping layer was assumed to be fully oxidized. Since the Heusler layers have higher resistivities than the CTLs the estimated current density is an upper limit.

### Code availability

The code that supports the findings of this study is available from the corresponding author upon reasonable request.

## Electronic supplementary material

Supplementary Information

## Data Availability

The authors declare that the data supporting the findings of this study are available within the paper and its supplementary information files.
