# Supplementary material for: Chiral domain wall motion in unit-cell thick perpendicularly magnetized Heusler films prepared by chemical templating
Source: Nat Commun. 2018 Nov 7;9:4653. doi: 10.1038/s41467-018-07091-3 (PMC6220290; doi:10.1038/s41467-018-07091-3)
Supplement: Supplementary file 1 — Supplementary Information [file 41467_2018_7091_MOESM1_ESM.pdf]

**Supplementary Information**

**Chiral domain wall motion in unit-cell thick perpendicularly magnetized Heusler films prepared by chemical templating**

Filippou *et al.*

**Supplementary Note 1. Chemical templating layer (CTL) candidates**

The candidate materials for the CTL are listed in Supplementary Table 1. These materials have a B2 structure with a small lattice mismatch (< 7%) compared to the in-plane lattice parameter of tetragonal $Mn_3Sn$ found in [1]. Lattice constants are obtained from Pearson's crystal database.

| Type | X'Y' | Lattice Constant [A] | Mismatch with $Mn_3Sn$ [%] |
|---|---|---|---|
| Intermetallic | NiAl | 2.89 | 4.0 |
| Intermetallic | FeAl | 2.91 | 4.5 |
| Intermetallic | CoAl | 2.86 | 2.8 |
| Intermetallic | RhAl | 2.97 | 6.4 |
| Intermetallic | ReAl | 2.88 | 3.5 |
| Intermetallic | CoGa | 2.88 | 3.5 |
| Intermetallic | NiGa | 2.88 | 3.5 |
| Intermetallic | MnNi | 2.97 | 6.4 |
| Intermetallic | MnV | 2.94 | 5.5 |
| Intermetallic | FeTi | 2.98 | 6.7 |
| Intermetallic | CuZn | 2.95 | 5.8 |
| Intermetallic | BeTi | 2.94 | 5.5 |
| Intermetallic | CoSn | 2.98* | 6.7 |
| Intermetallic | CoGe | 2.71* | -2.6 |
| Silicide | CoSi | 2.82 | 1.4 |
| Silicide | OsSi | 2.96 | 6.1 |
| Silicide | RuSi | 2.91 | 4.5 |
| Fluoride | AgF | 2.94 | 5.5 |

**Supplementary Table 1**. List of CTL candidates with a B2 structure and a lattice constant between 2.8 and 3Å. *Experimental value obtained from X-ray diffraction (XRD) out of plane lattice constant measurement, for a tetragonal structure.

**Supplementary Note 2. Chemical ordering and morphological properties of the (CoZ') CTLs**

XRD measurements were carried out at room temperature using a Bruker General Arrea Detector Diffraction System (GADDS) with a Cu Kα X-ray source for the CoAl, CoGa, CoSn and CoGe CTLs that were annealed at various temperatures ($T_{AN}$). The annealing duration was 30 min in all cases. XRD data for CoAl CTLs shown in Supplementary Fig. 1 show presence of both (001) and (002) peaks in all cases. The existence of the CoAl (001) superlattice peak clearly proves that there is an alternate layering of Co and Al atoms for all annealing temperatures and even without any annealing. The low intensity observed for the MgO (002) substrate peak is due to the step size limitation of the X-ray area detector. High resolution XRD measurements for the CoAl CTL grown at room temperature and without any anneal are shown in Supplementary Fig. 2. The $\theta$-2$\theta$ scan, phi scan of MgO (202) and CoAl (101) and reciprocal space mapping of CoAl (101) were measured on a Bruker D8 discover high resolution XRD system. The data shows an excellent epitaxy of the CoAl film with the MgO substrate with in-plane relationship MgO[100]//CoAl[110]. We find no evidence of presence of a second phase in the CoAl CTL.

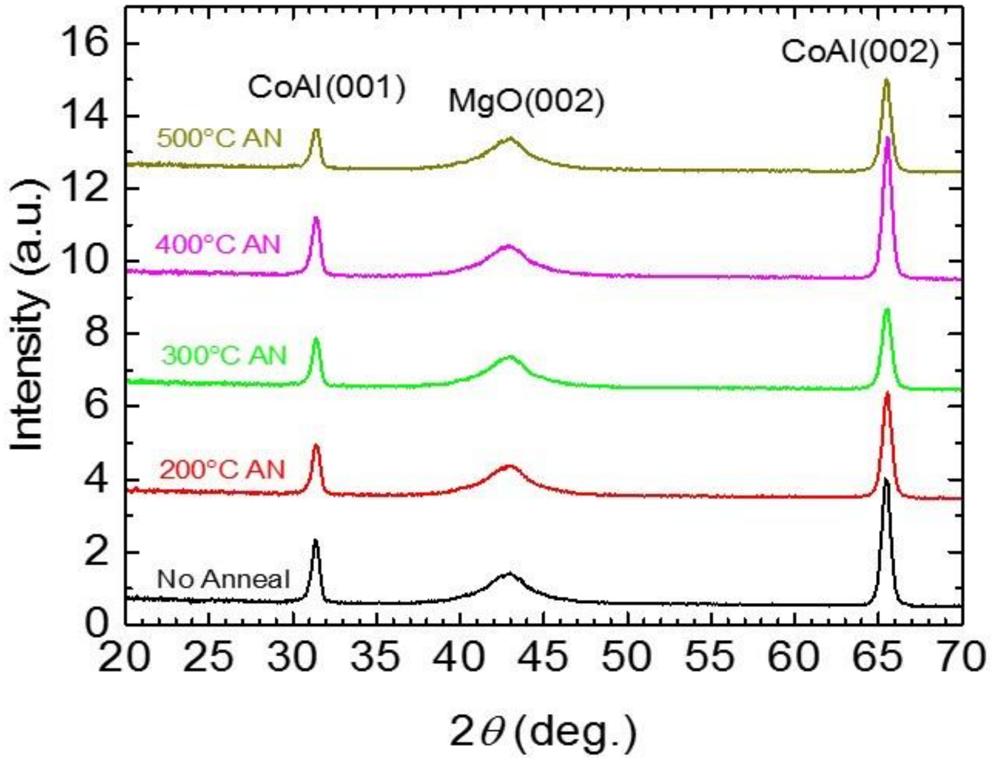


**Supplementary Figure 1**. XRD $\theta$-$2\theta$ curves of 300 Å thick CoAl films on MgO (001) | 20 Å MgO. The CoAl layers were deposited at ambient temperature and annealed at various $T_{AN}$. The films were capped with 20 Å MgO | 30 Å Ta to prevent ambient oxidation.

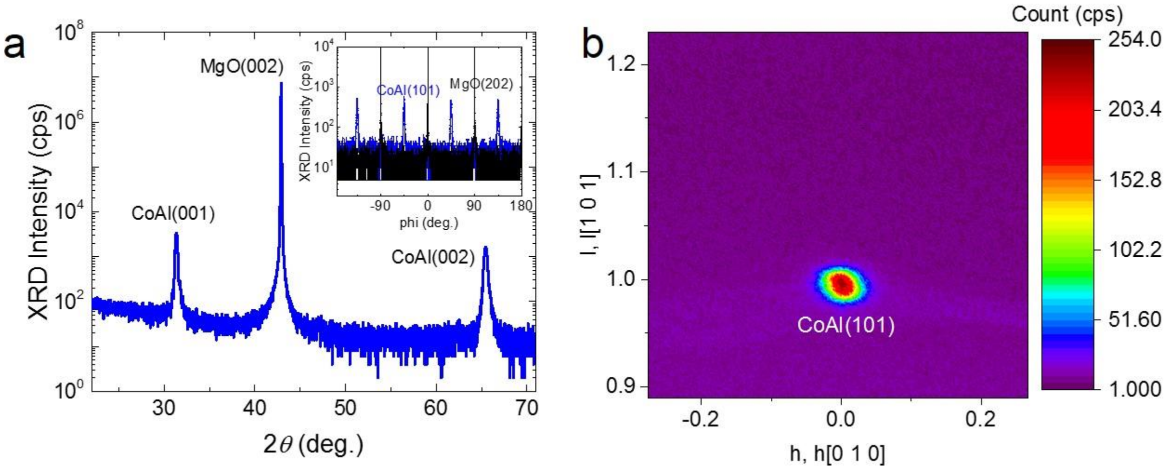


**Supplementary Figure 2**. XRD data of a 300 Å thick CoAl film on MgO (001) | 20 Å MgO. The CoAl layer was deposited at ambient temperature without any post annealing and capped with 20 Å MgO | 30 Å Ta. (a) XRD $\theta$-2$\theta$ scan, (a, inset) phi scan, and (b) reciprocal space mapping of CoAl (101) peak.

In Supplementary Fig. 3 we include XRD data for the CoGa CTLs measured on a Bruker GADDS system. Both (001) and (002) peaks were observed and the existence of the CoGa (001) superlattice peak indicates the alternate layering of Co and Ga atoms. The intensity of (001) peak increases with annealing reflecting an improved ordering within the CoGa layer at higher anneal temperatures.

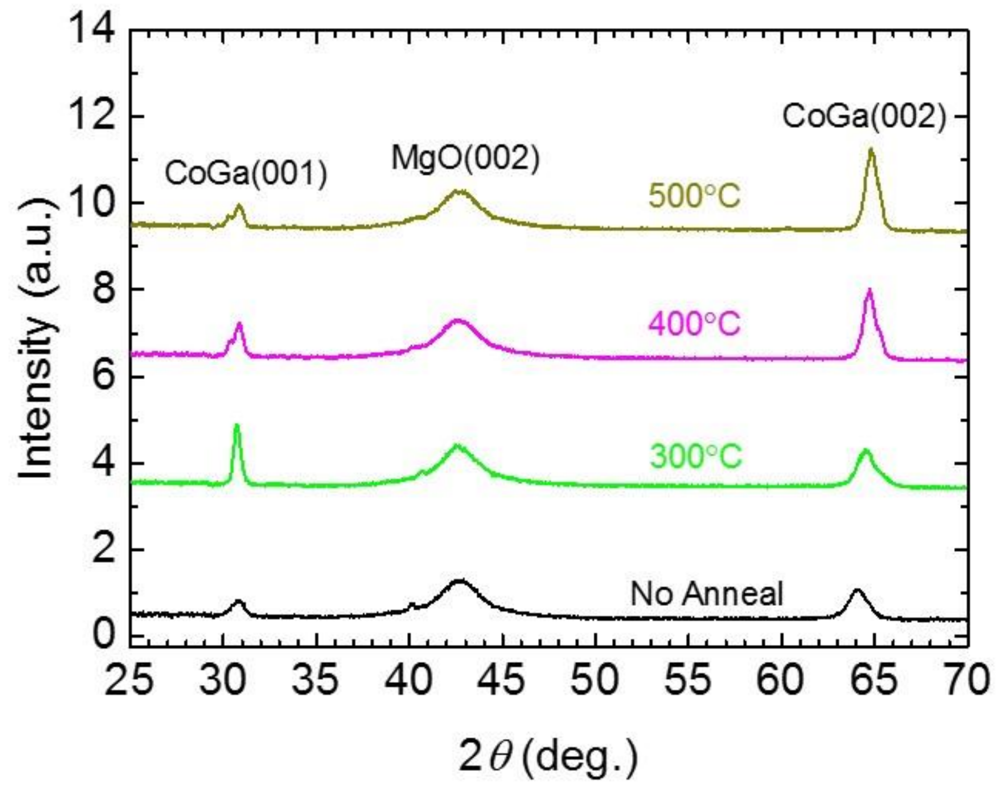


**Supplementary Figure 3**. XRD $\theta$-$2\theta$ curves of 300 Å thick CoGa films on MgO (001) | 20 Å MgO. The CoGa layer were deposited at ambient temperature and annealed at various $T_{AN}$. The films were capped with 20 Å MgO | 30 Å Ta to prevent ambient oxidation.

Similarly, in Supplementary Fig. 4, XRD scans on CoSn CTLs show (001) and (002) peaks but only for low anneal temperatures. Both (001) and (002) peaks disappear after annealing at $T_{AN} \geq$ 400 $^{o}$C. The surface roughness of the CoSn CTL also increases dramatically at high anneal temperatures (see Fig. 1e).

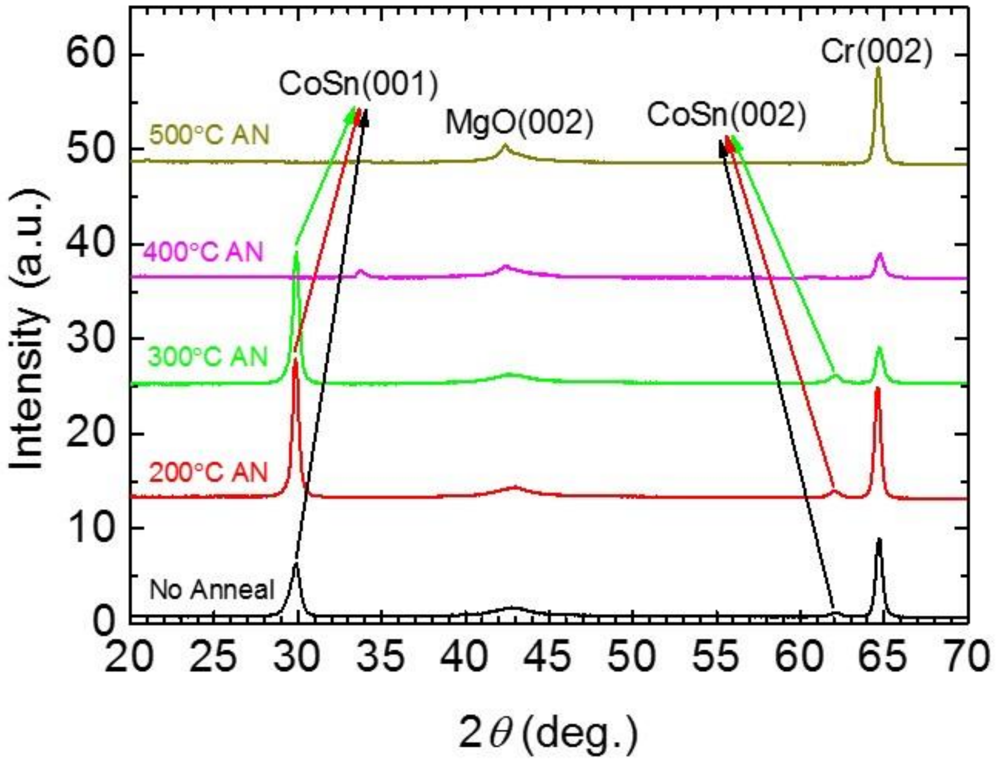


**Supplementary Figure 4**. XRD $\theta$-2$\theta$ curves of 300 Å thick CoSn films on MgO (001) | 20 Å MgO | 400 Å Cr seed layer. The CoSn layer were deposited at ambient temperature and annealed at $T_{AN}$. The films were capped with 20 Å MgO | 30 Å Ta to prevent ambient oxidation.

Another important parameter is the surface roughness of the CTLs. We find that the root-mean-square roughness ($r_{rms}$), as determined by atomic force microscopy, depends strongly on $T_{AN}$ for certain compositions of the CoGa CTL. The films with the preferred $Co_{53}Ga_{47}$ composition show the smoothest surface (see Supplementary Fig. 5).

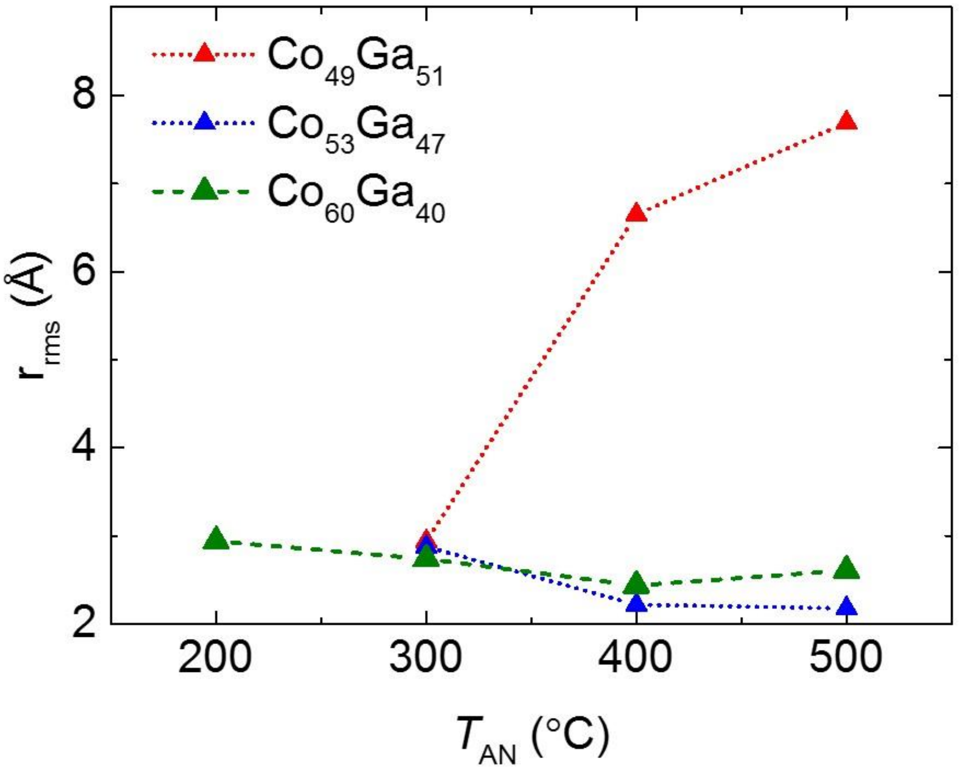


**Supplementary Figure 5**. Root-mean-square roughness versus annealing temperature for various $Co_xGa_{1-x}$ compositions. For the optimum composition, $Co_{53}Ga_{47}$, the films remain atomically smooth (~2 Å) even after annealing at high $T_{AN}$.

Electron energy loss spectroscopy (EELS) line scans performed on XTEM micrographs of the CoGa CTLs display the relative concentration of various elements in the film stack with atomic resolution. In Supplementary Fig. 6 the concentration profiles for Co and Ga elements for a CoGa CTL oscillate, such that the maximum intensity of one is at a minimum intensity of the other, clearly indicating the presence of alternating atomic layers of Co and Ga. These EELS spectra were acquired with a 7C probe size (nominal current 70 pA), in 1 Å steps at 0.05-0.005 sec/pixel exposure times.

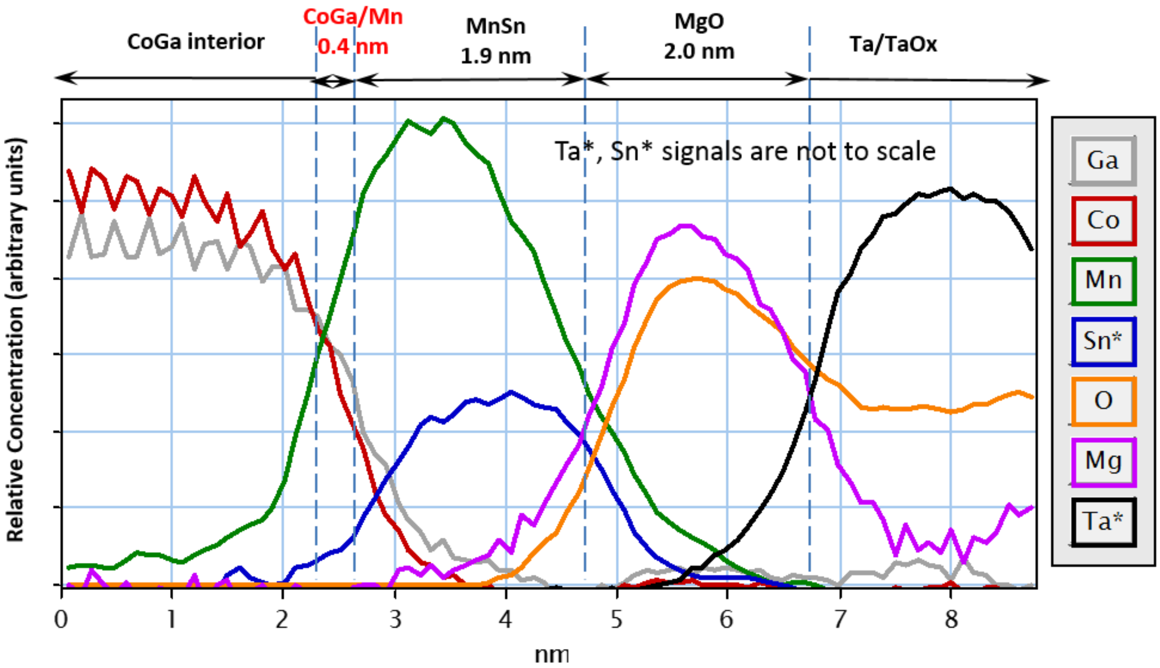


**Supplementary Figure 6**. EELS profiles of the elements indicated for a structure with a CoGa CTL of the form: MgO (001) | 20 Å MgO | 400 Å Cr | 300 Å CoGa | 20 Å $Mn_3Sn$ | 20 Å MgO | 30 Å Ta, for $T_{AN}$ = 500 $^oC$.

Furthermore, we show in Supplementary Fig. 7 that a CoGe CTL forms alternating layers exhibiting (001) and (002) superlattice peaks for $T_{AN}$ = 500 °C. Perpendicular magneto-optic Kerr Effect (P-MOKE) hysteresis loops for MnGa films on CoGe CTLs have PMA only when there is atomic ordering within the CTL.

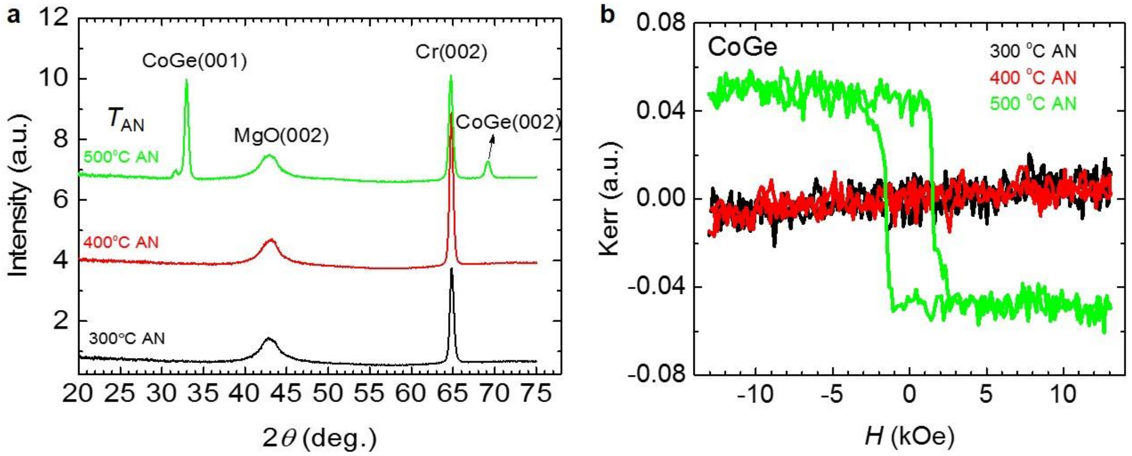


**Supplementary Figure 7**. (a) XRD $\theta$-$2\theta$ curves of a CoGe CTL within the film stack of MgO (001) | 20 MgO | 400 Cr | 300 CoGe | 20 MnGa | 20 MgO | 30 Ta (thicknesses in Å). The CoGe layer was deposited at ambient temperature and annealed at $T_{AN}$. (b) Corresponding P-MOKE hysteresis loops.

## Supplementary Note 3. $Mn_3Z$ Heusler tetragonal structures

The ultra-thin Heusler films grown on the CTL, form alternating layers of Mn-Mn and Mn-Z layers. This is clearly seen in the STEM images included in Fig. 2 of the main text. In Supplementary Fig. 8 we show the conventional unit cell of the $Mn_3Z$ structure. The arrows on the Mn atoms indicate the orientation of their magnetic moment.

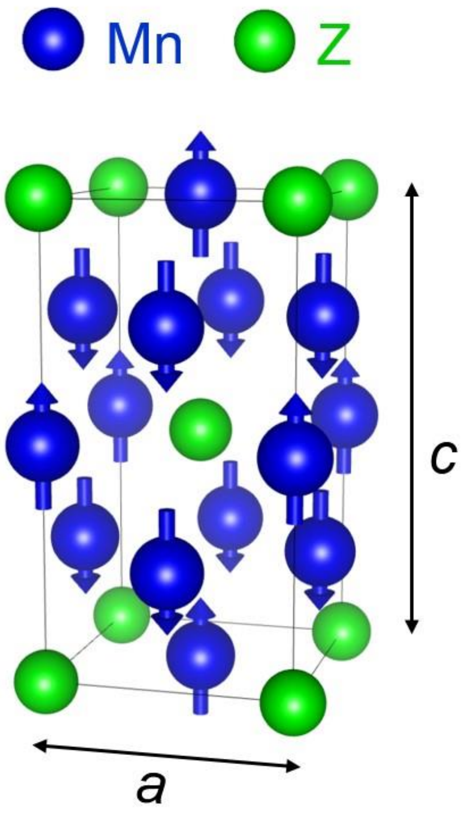


**Supplementary Figure 8**. $D0_{22}$-tetragonal unit cell of $Mn_3Z$ (Z = Ge, Sn, Sb) Heusler. The blue and green atoms correspond to Mn and Z atoms, respectively, *a* and *c* correspond to the in-plane and out of plane lattice constants, respectively, with $c > a$.

**Supplementary Note 4. Kerr microscopy in differential mode for imaging magnetic domains and DW motion**

In Supplementary Fig. 9a, we illustrate the domain wall (DW) motion observed using a Kerr microscope in differential mode. The position of the DW is indicated by a dotted line. In Supplementary Fig. 9b, the same images are overlaid with the magnetization direction. The initial magnetic state of the wire is taken at $t_0$ and subtracted from the subsequent images taken at $t_n$. Application of current pulses $t_1$ through $t_4$ move the DW to the right and this appears as an expansion of the domain in the differential Kerr image. The DW velocity was calculated from measurements of the expansion of the magnetic domain.

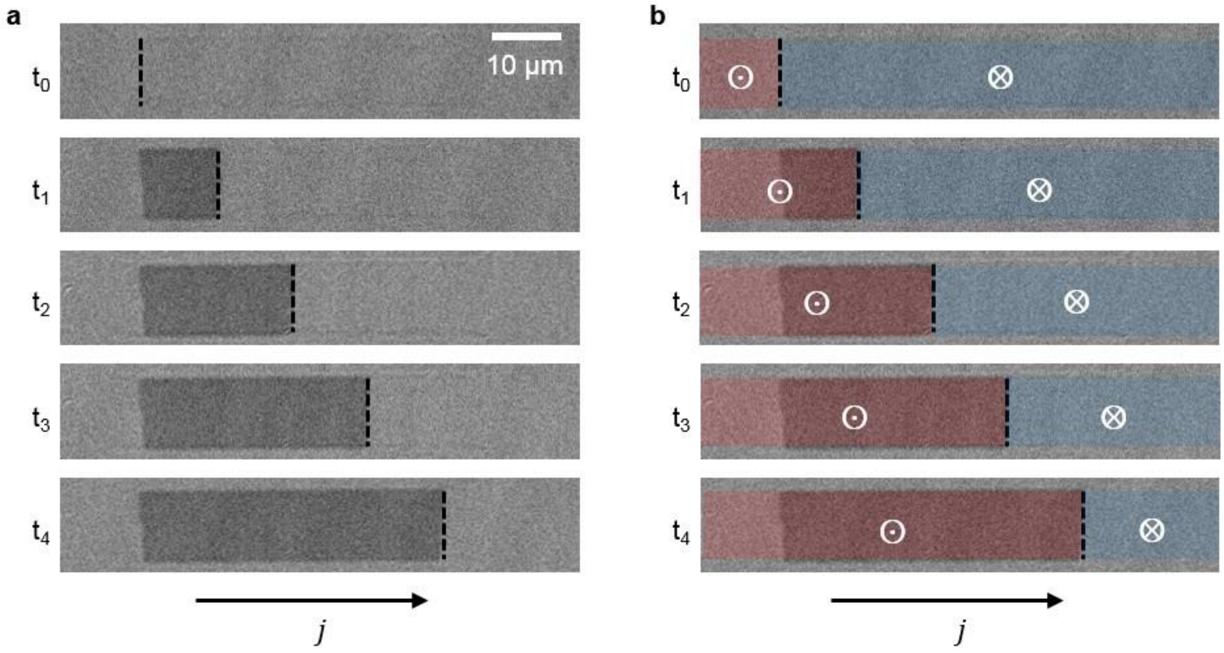


**Supplementary Figure 9.** DW motion for a nanowire imaged with differential Kerr microscopy. The wire is imaged from top and ↑ magnetization (dark contrast) is indicated by ⊙ while ↓ magnetization (bright contrast) is indicated by ⊗. **(a)** Kerr images with a dotted line showing the DW position. At $t_0$ no current is applied and the image represents the initial state which is subtracted from all subsequent images. **(b)** Same Kerr images from (a), overlaid with the corresponding magnetization directions within the domains.

**Supplementary Note 5. DW velocity versus *J* for different wire widths**

The DW velocity for different wire widths was measured. The DW motion is stable and identical for all wire widths. The voltage limits of our power supply were +50V and -45Vso that the width of the nanowire limited the maximum possible current density, $J$. The highest $J$ that could be achieved in the narrowest 2 μm wide nanowires is what determines the maximum current density shown in Supplementary Fig. 10 for various nanowires of different widths. These data clearly show that the DW velocity is independent of the nanowire width.

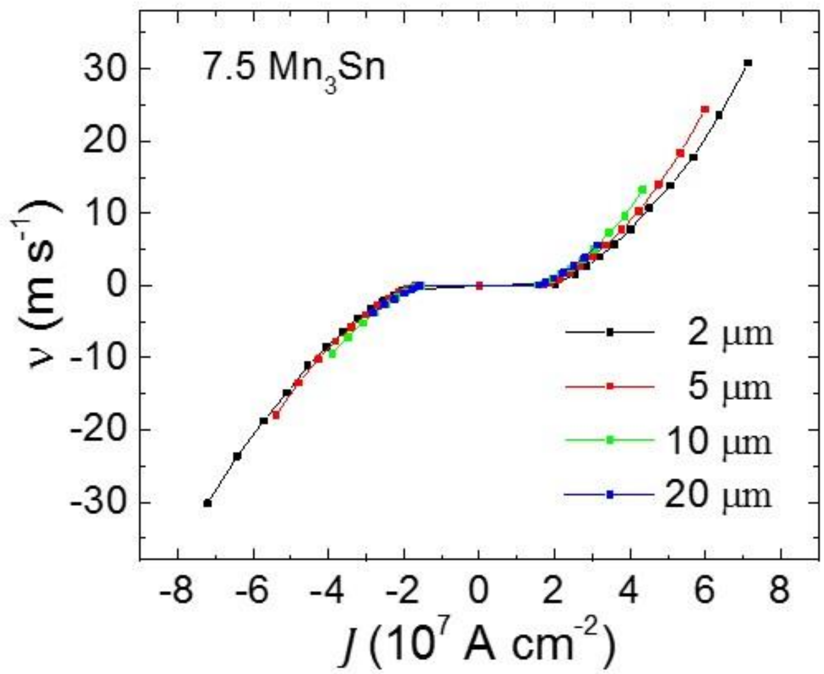


**Supplementary Figure 10**. DW velocity vs applied current density, $J$, for different wire widths of 2, 5, 10, 20 μm for a 7.5 Å $Mn_3Sn$ Heusler film. The current pulse length used was 100 ns.

## Supplementary Note 6. DW velocity versus $H_x$, $H_y$ for CoAl | 20 Å $Mn_{2.7}Sb$ and CoSn | 20 Å $Mn_{2.0}Sb$

Supplementary Fig. 11 presents the corresponding $H_x$ and $H_y$ field dependence for the films shown in Fig. 3f. $H_x$ and $H_y$ are magnetic fields that are applied in the plane of the nanowire along and transverse to the nanowire, respectively.

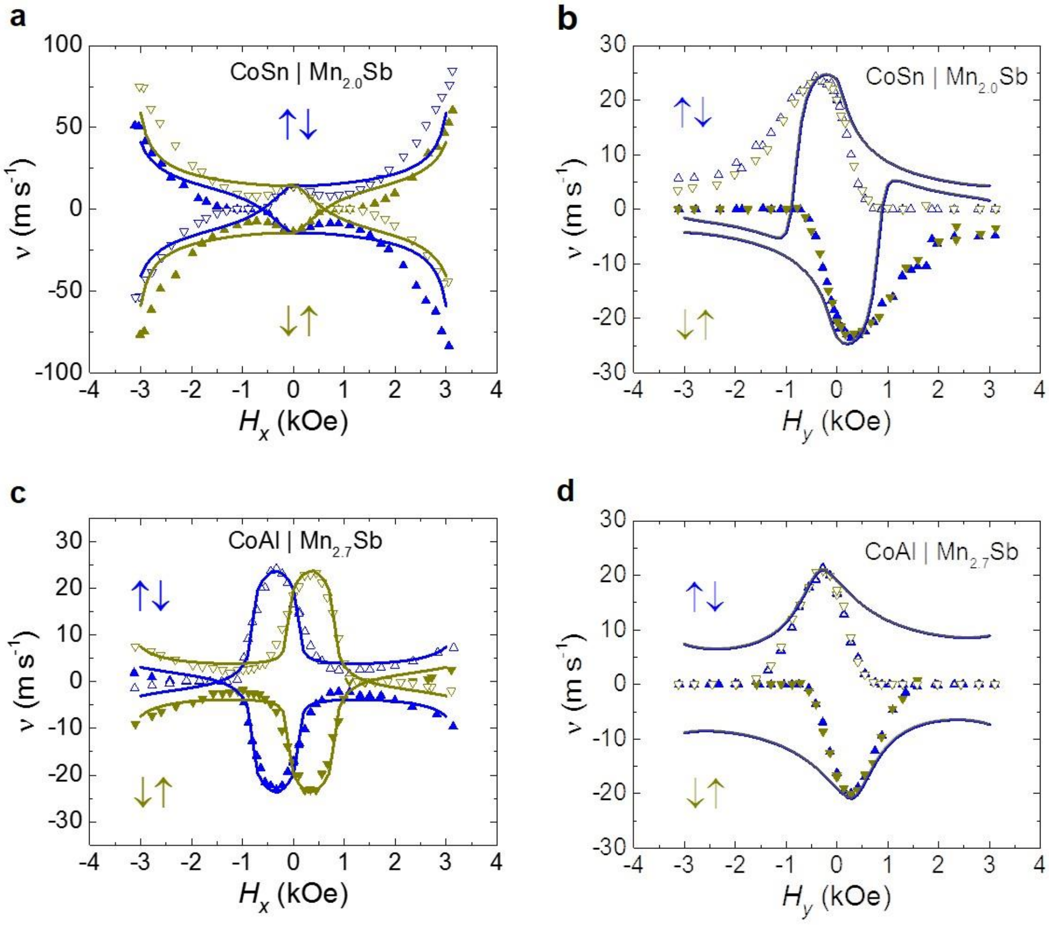


**Supplementary Figure 11**. **(a)**,**(b)** DW velocity, *v*, versus $H_x$ and $H_y$ field for ↑↓ (blue) and ↓↑ (dark yellow) DWs for the film stack: MgO (001) | 20 MgO | 30 CoGa | 20 CoSn | 20 $Mn_{2.0}Sb$ | 20 MgO | 20 Ta. Current densities, $J$, of $\sim 5.2 \times 10^7$ and $\sim 6.6 \times 10^7$ A $cm^{-2}$ were used for (a) and (b) respectively. **(c)**,**(d)** DW velocity, *v*, versus $H_x$ and $H_y$ field for ↑↓ (blue) and ↓↑ (dark yellow) DWs for the film stack: MgO (001) | 20 MgO | 50 CoAl | 20 $Mn_{2.7}Sb$ | 20 MgO | 20 Ta. Current densities, $J$, of $\sim 5.4 \times 10^7$ and $\sim 5.6 \times 10^7$ A $cm^{-2}$ were used for (c) and (d) respectively. Positive and negative currents are shown with closed and open symbols, respectively. All thicknesses are in Å. Solid lines represent the 1D model simulation.

**Supplementary Note 7. DW velocity versus $H_x$, $H_y$ for 50 Å CoGa CTL | 7.5 Å $Mn_3Sn$ with and without a 50 Å CoGa capping layer.**

Supplementary Fig. 12 shows a comparison of the dependence of DW velocity on $H_x$ and $H_y$ for both ↑↓ and ↓↑ DWs for the case of an MgO capping layer (**a** and **b**) and with a capping layer identical to the CTL (**c** and **d**). Note that Fig. 5c and d of the main text present data only for ↑↓ DWs.

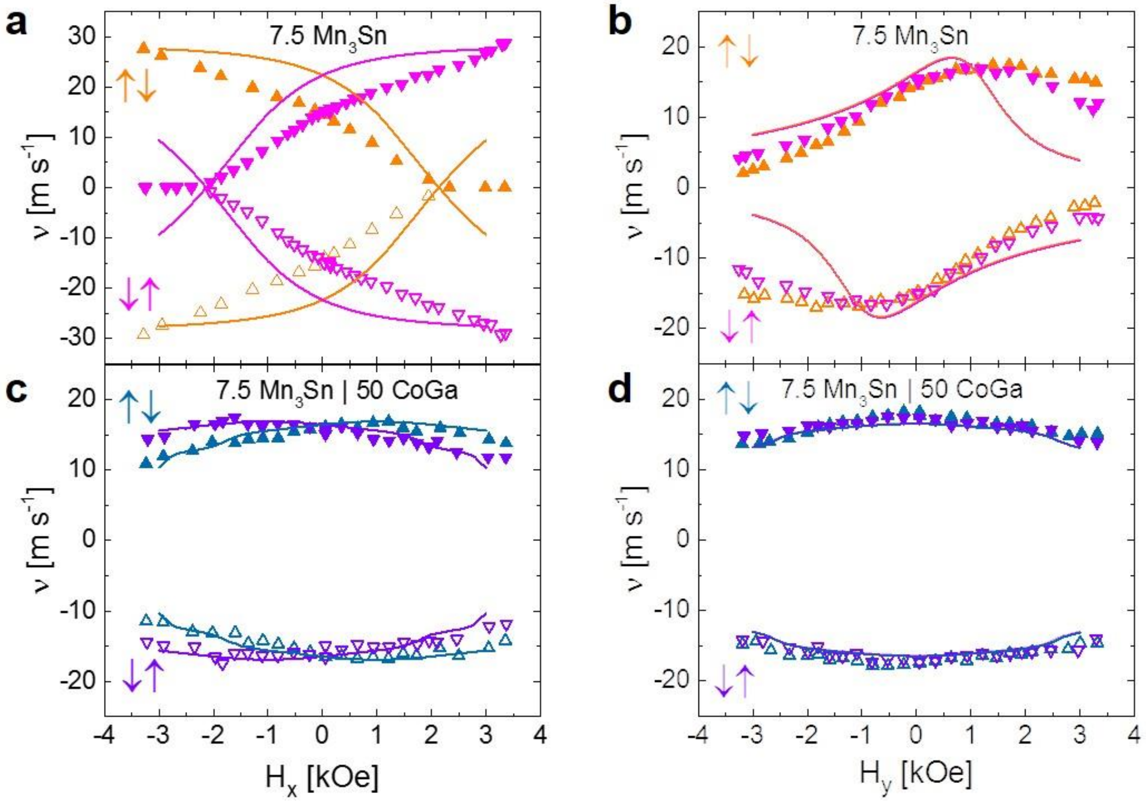


**Supplementary Figure 12**. ↑↓ and ↓↑ DW velocity versus in-plane fields, $H_x$ and $H_y$, for 7.5 Å $Mn_3Sn$, without CoGa overlayer **(a)**, **(b)** and with a CoGa overlayer **(c)**, **(d)**. In the case of no CoGa overlayer, the stack is: MgO (001) | 20 MgO | 50 CoGa | 7.5 $Mn_3Sn$ | 20 MgO | 20 Ta. With CoGa overlayer the stack is: MgO (001) | 20 MgO | 50 CoGa | 7.5 $Mn_3Sn$ | 50 CoGa | 20 MgO | 20 Ta (all thicknesses in Å). Closed and open symbols represent positive and negative applied currents, respectively. Current densities, $J$, of $\sim 5.0 \times 10^7$ A cm$^{-2}$ for (**a**) and (**b**) and $\sim 5.7 \times 10^7$ A cm$^{-2}$ for (**c**) and (**d**) were used, respectively. Solid lines represent the 1D model simulations. Note that in the $H_y$ field dependence, the simulated values for ↑↓ and ↓↑ DW configurations overlap.

**Supplementary Note 8. DW velocity versus $H_x$, $H_y$ for $Mn_3Sn$ layer with variable thickness: t=7.5, 10, 15 Å**

We show the DW velocity dependence on in-plane field for various thicknesses of $Mn_3Sn$. Both ↑↓ and ↓↑ DW configurations are shown in each graph.

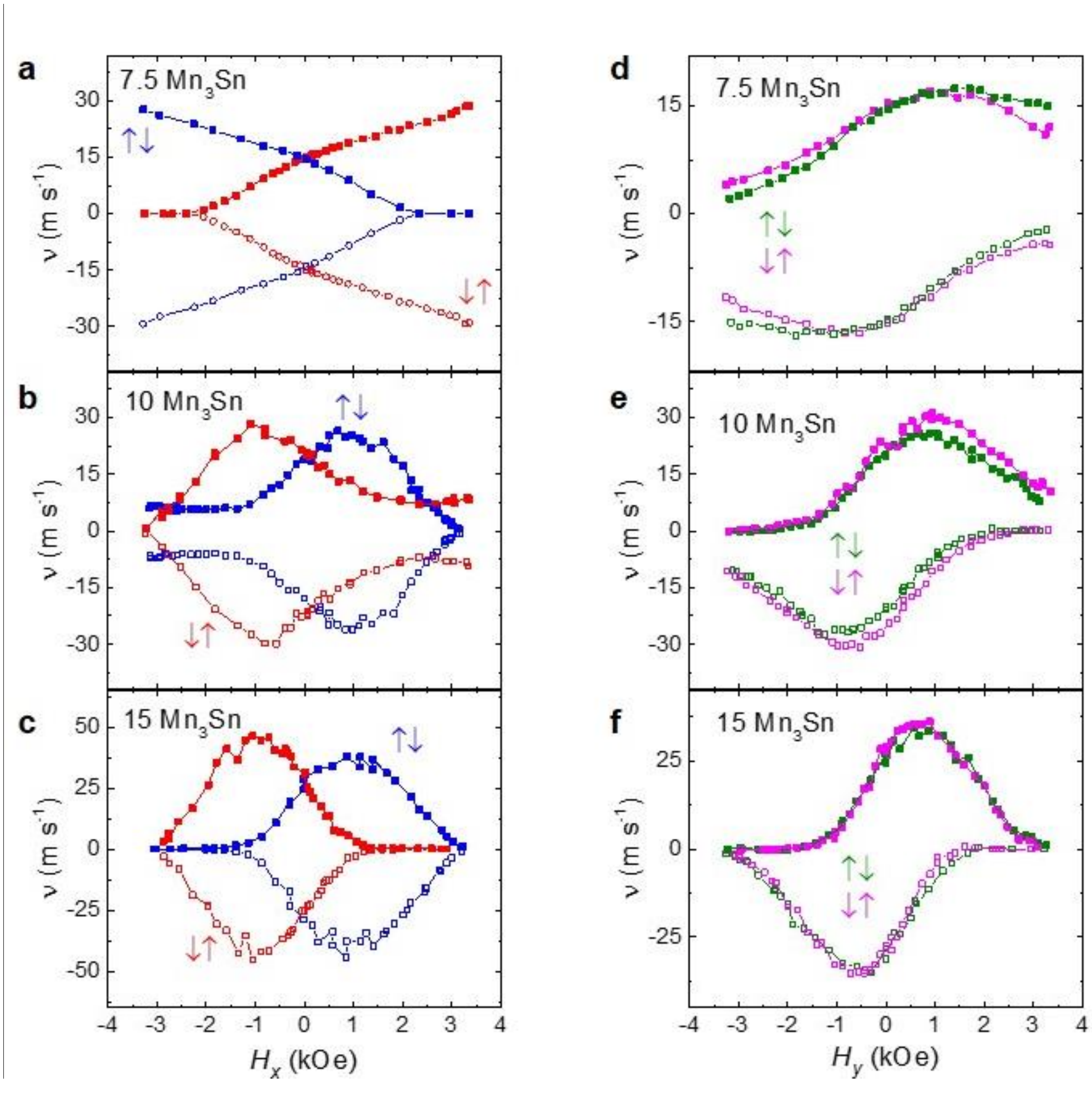


**Supplementary Figure 13**. DW velocity, *v*, versus longitudinal $H_x$ field for ↑↓ (blue) and ↓↑ (red) DWs. Positive and negative currents are shown with closed and open symbols respectively. t = 7.5 **(a)**, 10 **(b)**, 15 **(c)**, in Å. *v* versus transverse $H_y$ field for ↑↓ (green) and ↓↑ (magenta) DWs. Positive and negative currents are shown with closed and open symbols respectively. t = 7.5 **(d)**, 10 **(e)**, 15 **(f)**, in Å. Current densities, $J$, of $\sim 7.5 \times 10^7$, $\sim 8.6 \times 10^7$ and $\sim 9.1 \times 10^7$ A $cm^{-2}$ were used for the 7.5, 10 and 15 Å thick $Mn_3Sn$, respectively.

**Supplementary Note 9. DW velocity dependence on pulse length for Heusler films with different thicknesses**

The DW velocity dependence on the Heusler film thickness for various current pulse lengths was explored. Furthermore, we compare the critical current density for DW motion, $J_c$, for current pulse lengths varying from 5 ns for $Mn_3Sn$ and 20 ns for $Mn_3Ge$ to 100 ns for all thicknesses. The decreasing resistivity with increasing Heusler layer thickness can account for this dependence. For thicker films, the DW motion becomes insensitive to the pulse length.

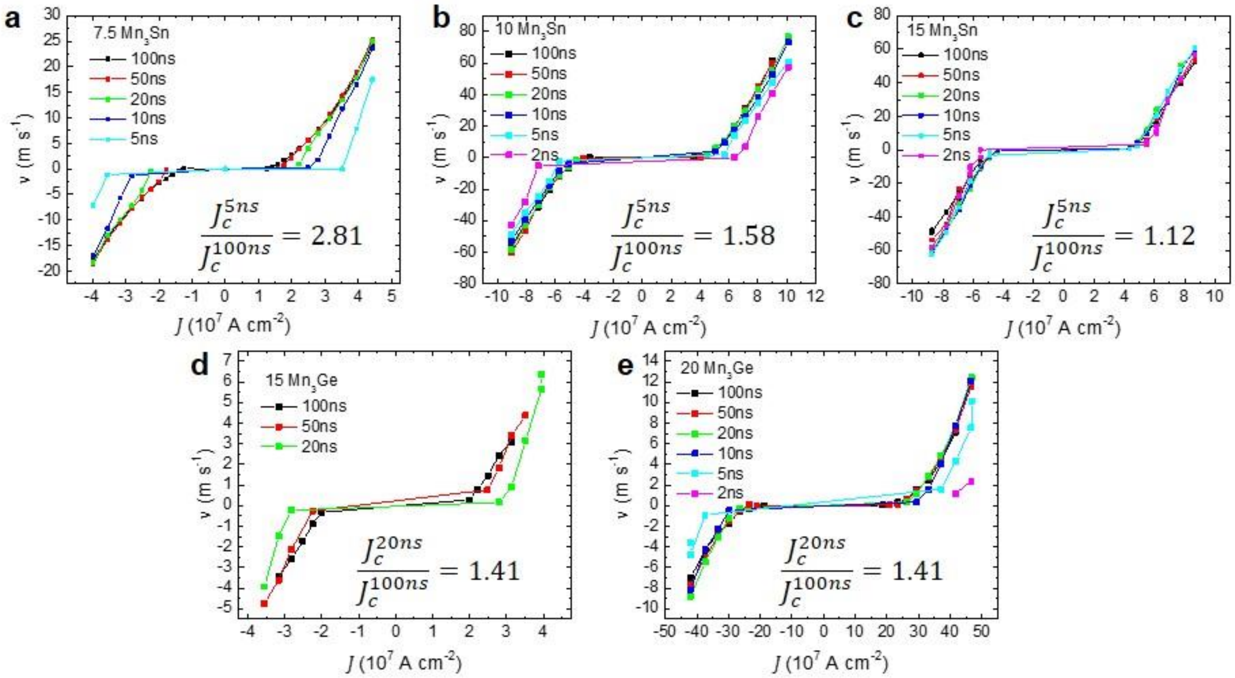


**Supplementary Figure 14**. Dependence of critical current density on current pulse length for 7.5, 10, 15 Å $Mn_3Sn$ **(a)-(c)** and 15-20 Å $Mn_3Ge$ **(d)-(e)**.

**Supplementary Note 10. SHE dependence on the overlayers: W, Pt**

The DW motion in ultra-thin Heusler can be strongly influenced by capping layers that show high Spin Hall Angle (SHA). Fig. 5e showed the DW velocity versus current density and in Supplementary Fig. 15 we present the DW velocity versus $H_x$ and $H_y$. When we insert a W overlayer (blanket film resistivity ρ=173 μΩ cm) which has a negative sign SHA (see main text and Supplementary Table 3 for $\theta_{\mathrm{SH}}$), the spin orbit torques are enhanced and this can be seen by the high slope observed in the DW velocity versus $H_x$ field dependence (Supplementary Fig. 15a). When a Pt overlayer is introduced, the SHA is of the same sign as the CoGa CTL, thus suppressing the spin orbit torques. As the Pt overlayer thickness is increased from 5 to 10 Å Pt, if we compare Supplementary Fig. 15d and f, we see that the SHA reverses. This can be observed by the change of the asymmetry in the $H_y$ field dependence (see also Supplementary Table 3).

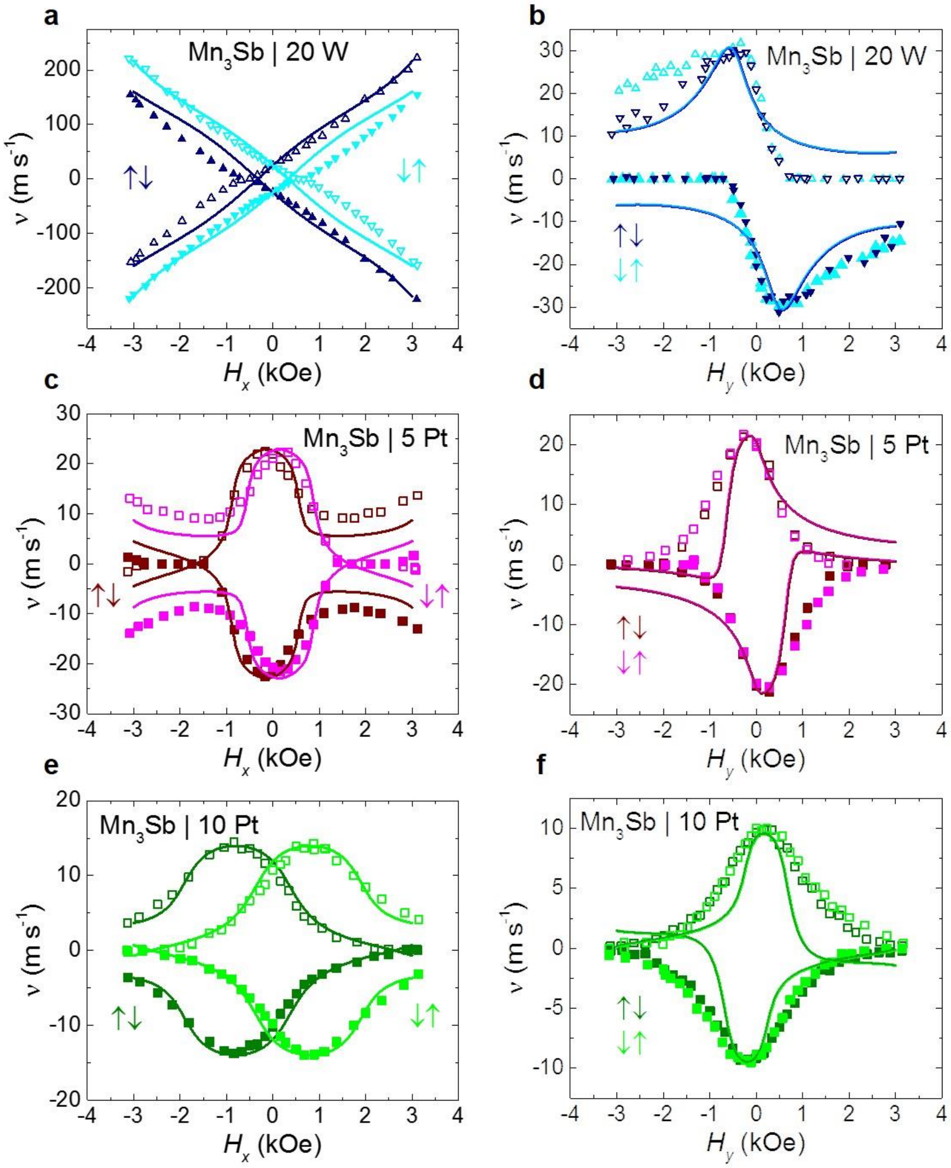


**Supplementary Figure 15**. ↑↓ and ↓↑ DW velocity vs longitudinal and trasnverse in-plane fields $H_x$, $H_y$ respectively. Solid lines represent the 1D model simulation. **(a)**, **(b)** show data for a W overlayer, for which the film stack was: MgO (001) | 20 MgO | 50 CoGa | 20 $Mn_3Sb$ | 5 CoGa | 20 W | 20 MgO | 20 Ta with current densities, $J$, of $\sim 3.4 \times 10^7$ and $\sim 3.5 \times 10^7$ A $cm^{-2}$ respectively. **(c)**, **(d)** show data for a 5 Å Pt overlayer for which the film stack was: MgO (001) | 20 MgO | 50 CoGa | 20 $Mn_3Sb$ | 5 CoGa | 5 Pt | 20 MgO | 20 Ta with current densities, $J$, of $\sim 7.4 \times 10^7$ and $\sim 7.2 \times 10^7$ A $cm^{-2}$ respectively. **(e)**, **(f)** show data for a 10 Å Pt overlayer for which the film stack was: MgO (001) | 20 MgO | 50 CoGa | 20 $Mn_3Sb$ | 5 CoGa | 10 Pt | 20 MgO | 20 Ta with current densities, $J$, of $\sim 7.2 \times 10^7$ and $\sim 7.0 \times 10^7$ A $cm^{-2}$ respectively. All thicknesses are in Å.

**Supplementary Note 11. $H_x$ dependence of DW velocity and 1D model simulations for $Mn_xSb$**

The fits of the 1D model to the DW velocity data presented in Fig. 5f of the main manuscript are presented below.

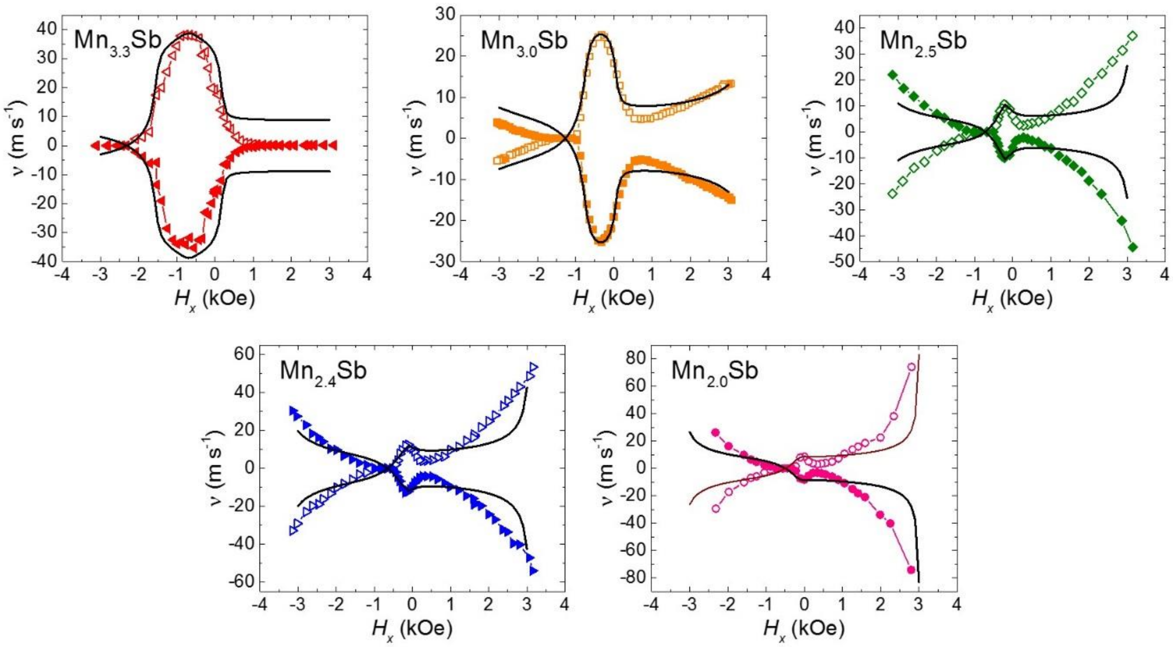


**Supplementary Figure 16**. ↑↓ DW velocity vs longitudinal field $H_x$ with fits from the 1-D model (solid black lines). Closed and open symbols represent positive and negative currents, respectively.

**Supplementary Note 12. *$H_y$* dependence of DW velocity and 1D model simulation for $Mn_xSb$**

The corresponding $H_y$ field dependence of DW velocity and the 1D model simulations for $Mn_xSb$ films are shown in Supplementary Fig. 17.

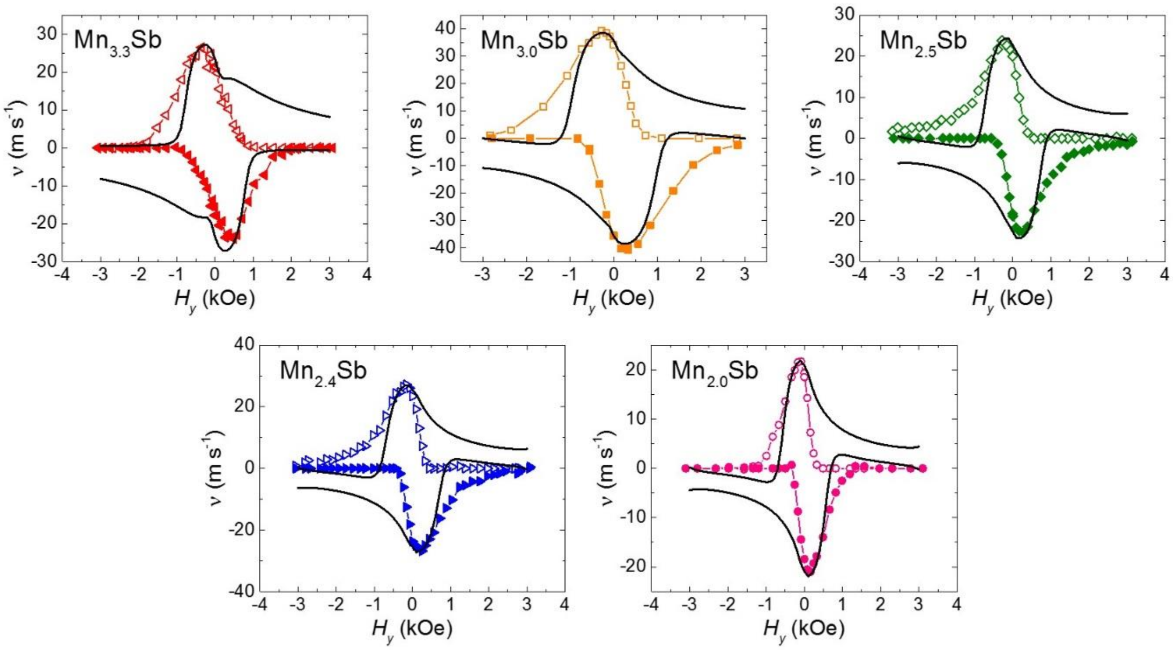


**Supplementary Figure 17**. ↑↓ DW velocity vs in-plane transverse field $H_y$ and fit from the 1-D model (solid black lines). Closed and open symbols represent positive and negative currents, respectively.

**Supplementary Note 13. DMI exchange field in the $Mn_xSb$ nanowires**

The Dzyaloshinskii-Moriya interaction (DMI) exchange field determined by 1D model fits to the $H_x$ field dependence data shown in Supplementary Fig. 16 as a function of composition of $Mn_xSb$ are summarized in Supplementary Fig. 18.

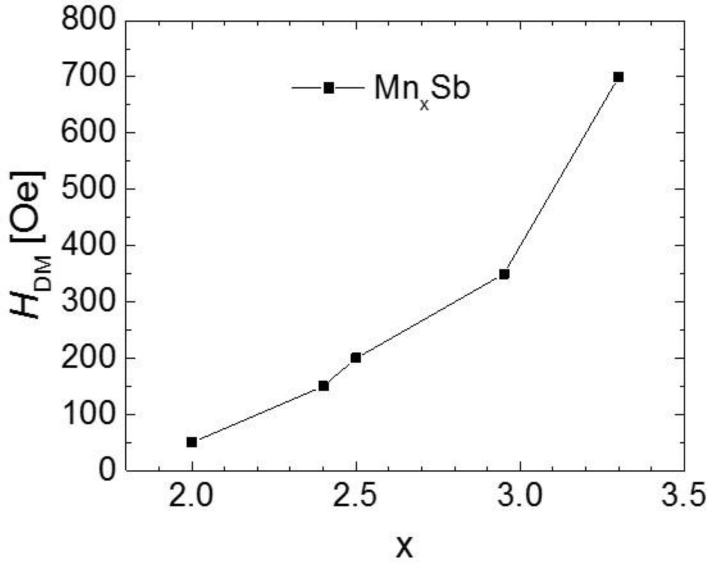


**Supplementary Figure 18**. $H_{DM}$, determined by fitting the1D model to the DW velocity dependence on longitudinal in-plane field versus the composition of several $Mn_xSb$ films.

**Supplementary Note 14. Comparison of P-MOKE hysteresis loops for Heusler films grown at 30 °C (room temperature) and 100 °C on CoGa CTLs**

We show a comparison of magnetic properties of 20 Å $Mn_3Ge$ films deposited at 30 °C and at 100 °C on a CoGa CTL. Magnetic properties of these Heusler films are very similar and hence in the current study, ambient deposition temperatures refers to samples deposited in the temperature range between 30 and 100 °C.

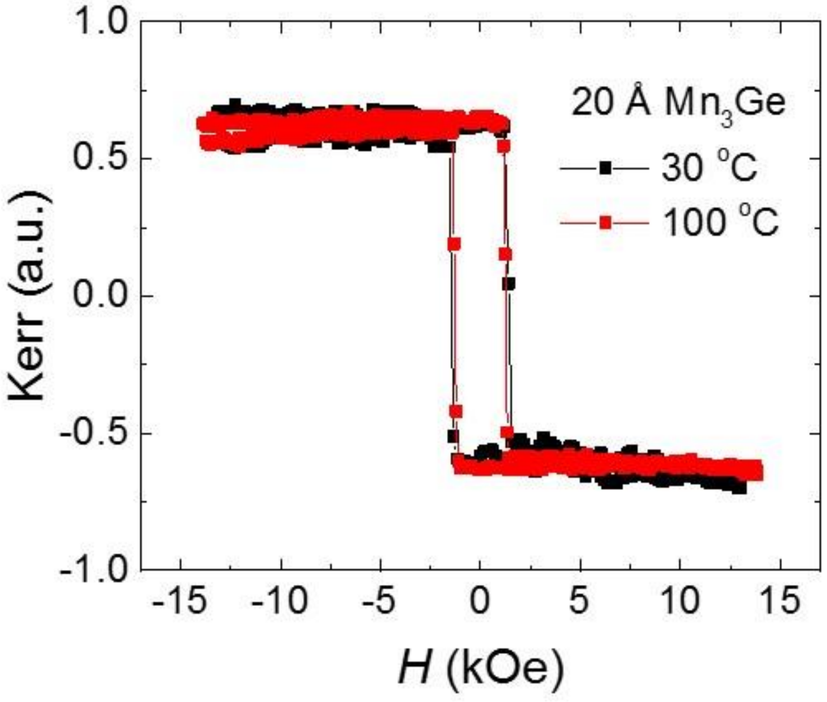


**Supplementary Figure 19**. P-MOKE hysteresis loops for $Mn_3Ge$ Heusler films grown at 30 °C and 100 °C on a CoGa CTL.

**Supplementary Note 15. One-dimensional analytical model and DW motion mechanism**

The one-dimensional model [2,3] provides not only insights but also a quantitative understanding of the current driven domain wall dynamics. This model has been successful in describing the current induced motion of DWs in straight racetracks formed from single magnetic layers with perpendicular magnetic anisotropy (PMA) via a chiral spin torque [4,5], and in synthetic ferromagnets and antiferromagnets via an additional exchange coupling torque [6]. Here we use this model to investigate the current driven DW motion in nanowires formed from $Mn_3Z$ Heusler alloys. We model these ferrimagnetic Heuslers by a ferromagnetic layer whose magnetization is the net magnetization of the two sub-lattices in the ferrimagnet. This is a reasonable approximation when the two sub-lattices are strongly anti-ferromagnetically coupled. Indeed, as we show below, such a model well accounts for many details of the current induced domain wall motion in our Heusler films.

Note that for better illustrating the out of plane axes and torques, we will now refer to ↑ and ↓ as ⊙ and ⊗ respectively. Therefore, a ↑↓ DW configuration will be referred to as ⊙ | ⊗ and ↓↑ as ⊗ | ⊙.

The basic assumption of our model is that the DW has a fixed magnetization profile with the magnetization rotated from the direction perpendicular to the layer, i.e. the *z*-axis, by a polar angle $\theta$, and by the azimuthal magnetization angle $\psi$ which is defined in the plane of the wire with respect to the direction $\hat{x}$. Note that $\psi$ is constant and independent of the lateral position of the DW along the wire when it is manipulated either by current or magnetic field or a combination of the two (see Supplementary Fig. 20). For the case of perpendicularly magnetized nanowires, the DW dynamics can be described within the 1D model by two parameters, namely the position $q$ of the DW along the nanowire and its conjugate momentum $2M_s\psi/\gamma$. $M_s$ is the saturation magnetization and $\gamma$ is the gyromagnetic ratio. The DW motion in nanowires is governed by $\psi$, with the domain wall profile located at $\vec{r}$ and at time *t*, given by

$$\theta(\vec{r},t) = 2\arctan\exp\left(\pm\frac{x-q}{\Delta}\right) \quad (1)$$

Here the upper and lower signs correspond to the ⊙ | ⊗ and ⊗ | ⊙ domain magnetic configurations, respectively, and $\Delta$ is the domain wall width parameter.

First, we formulate the Lagrangians that include the adiabatic and non-adiabatic spin transfer torques (STTs), external field driven torques, the spin Hall current torque, and the Dzyaloshinskii-Moriya exchange field. The equations of motion are then derived by the Lagrange-Rayleigh equations [7].

With the DW profile function (1), the Lagrangian $\mathcal{L}$ in the nanowire that contains the magnetostatic potential energy, including anisotropy, DW kinetic energy and adiabatic spin-transfer torque is given by:

$$\mathcal{L} = \int \left( \mathcal{E} + \frac{M_s}{\gamma}\psi\dot{\theta}\sin\theta - \frac{uM_s}{\gamma}\psi\frac{d\theta}{dx}\sin\theta \right) dx$$
$$= \Delta\, M_s H_k \cos^2\psi \mp 2M_s H_z q - \pi\,\Delta\, M_s H_p \cos(\psi - \psi_H) - \pi\,\Delta\, M_s H_{DM} \cos\psi$$
$$\mp \frac{2wM_s}{\gamma}\phi(\dot{q} + u) \tag{2}$$

Here the upper and lower signs correspond to the ⊙ | ⊗ and ⊗ | ⊙ magnetic DW configurations, respectively. $\mathcal{E}$ is the magnetostatic energy density of the domain wall per unit area and is given by:

$$\mathcal{E} = \frac{M_s H_k}{2}\sin^2\theta\cos^2\psi - M_s H_z\cos\theta - M_s H_p \sin\theta\cos(\psi - \psi_H) - M_s H_{DM}\sin\theta\cos\psi \tag{3}$$

Eq. (2) can be rewritten as $\mathcal{L} = \sigma + \int \left[\frac{M_s}{\gamma}\phi\dot{\theta}\sin\theta - \frac{uM_s}{\gamma}\phi\frac{d\theta}{ds}\sin\theta\right] dx$ where $\sigma$ is the magnetostatic potential energy, $\sigma = \int \mathcal{E} dx$. $H_k$ is the magnitude of the in-plane anisotropy field derived from the shape anisotropy of the DW that favors a Bloch wall over a Néel wall, $H_z$ is the out-of-plane field, $H_p$ and $\psi_H$ are the in-plane magnetic field and its angle with respect to the $+\hat{x}$ direction, respectively. $H_{DM}$ is the Dzyaloshinskii–Moriya interaction exchange field at the DW whose direction is always perpendicular to the DW length direction, thereby favoring Néel type walls but its sign depends on the domain configurations establishing the chirality of the domain walls. The volume spin transfer torque due to the current flowing within the magnetic layer is parameterized by $u = \frac{\mu_B PJ}{eM_s}$, where $\mu_B$ is the Bohr magneton, *e* is the electron charge, *P* is the spin polarization of the current and $J$ is the current density in the magnetic layer. The dissipative function $\mathcal{F}$ that includes damping, non-adiabatic spin-transfer torque, and spin-orbit torque is given by:

$$\mathcal{F} = \int \frac{\alpha M_s}{2\gamma}\left[\left(\frac{d}{dt} - \frac{\beta u}{\alpha}\frac{d}{ds}\right)\vec{m} + \frac{\gamma}{\alpha}H_{SH}\vec{m}\times\hat{y}\right]dx = \frac{\alpha M_s}{2\gamma}\left(\frac{2\dot{q}^2}{\Delta} \pm \frac{2\pi\gamma}{\alpha}\dot{q}H_{SH}\cos\psi + \frac{4\beta u}{\alpha\Delta}\dot{q}\right) \tag{4}$$

Here again the upper and lower signs correspond to the ⊙ | ⊗ and ⊗ | ⊙ magnetic DW configurations, respectively, $\beta$ is the non-adiabatic STT coefficient and $\alpha$ is the Gilbert damping. The spin Hall effect (SHE) is parameterized by an effective field $H_{SH}$ in the center of the wire that is given by $H_{SH} = \frac{\hbar\theta_{SH}J_{UL}}{2eM_s t_H}$ where $\hbar$ is $\frac{h}{2\pi}$, $h$ is Planck's constant, $J_{UL}$ is the current density in the underlayer, $t_H$ is the thickness of the magnetic layer, and $\theta_{SH}$ is the spin Hall angle (or the effective spin Hall angle that describes the magnitude of the spin accumulation that the magnetic layer is subjected to). Note that the accumulated spin from the spin Hall is transverse to the applied current direction along the wire length $x$, i.e. the spin current is along the $\hat{y}$ direction.

Based on Lagrangians $\mathcal{L}$ and dissipated function $\mathcal{F}$ obtained above, the Lagrange-Rayleigh equations $\frac{\partial\mathcal{L}}{\partial X} - \frac{d}{dt}\left(\frac{\partial\mathcal{L}}{\partial\dot{X}}\right) + \frac{\partial\mathcal{F}}{\partial\dot{X}} = 0$ ($X = q$ and $\psi$) finally lead to the equations of motion when $H_z = 0$ as following:

$$\alpha\dot{q} \pm \Delta\dot{\psi} = \pm\gamma\Delta H_z - \beta u \mp \frac{\pi}{2}\gamma\Delta H_{SH}\cos\psi \tag{5a}$$

$$\dot{q} \mp \alpha\Delta\dot{\psi} = -u \pm \frac{\gamma\Delta}{2}\left[-H_k\sin 2\psi + \pi H_p\sin(\psi - \psi_H) + \pi H_{DM}\sin\psi\right] \tag{5b}$$

Here again the upper and lower signs correspond to the ⊙ | ⊗ and ⊗ | ⊙ domain magnetic configurations, respectively.

The eqs. (5a,b) can be rewritten as

$$(1+\alpha^2)\dot{q} = -(1+\alpha\beta)u \mp \gamma\Delta\left[\frac{H_k}{2}sin2\psi - \frac{\pi H_p}{2}\sin(\psi - \psi_H) - \frac{\pi H_{DM}}{2}\sin\psi + \frac{\alpha\pi H_{SH}}{2}\cos\psi\right]$$

(6a)

$$(1+\alpha^2)\dot{\psi} = \mp\frac{\beta-\alpha}{\Delta}u + \gamma\left[\frac{\alpha H_k}{2}sin2\psi - \frac{\alpha\pi H_p}{2}\sin(\psi-\psi_H) - \frac{\alpha\pi H_{DM}}{2}\sin\psi - \frac{\pi H_{SH}}{2}\cos\psi\right]$$

(6b)

(upper signs: ⊙ | ⊗ and lower signs: ⊗ | ⊙).

When the spin Hall torque is very small, i.e. $H_{SH} \approx 0$, from eqs. (6a,b) we obtain:

$$\dot{q} = -\frac{\beta}{\alpha}u \mp \frac{\Delta}{\alpha}\dot{\psi} \tag{7}$$

Eq. (6b) tells us about the steady state condition, i.e. $\dot{\psi} = 0$, such that:

$$\left(\frac{\beta}{\alpha}-1\right)u = \pm\Delta\gamma\left[\frac{H_k}{2}sin2\psi - \frac{\pi H_p}{2}\sin(\psi-\psi_H) - \frac{\pi H_{DM}}{2}\sin\psi\right]$$

(8)

For example, when $H_k = 0$ and $H_{DM} = 0$, if $|u| > \left|\frac{\pi\,\Delta\gamma H_p}{2}\frac{\alpha}{\beta-\alpha}\right|$ there exists no zero of $\psi$ to satisfy $u = \pm\frac{\pi\Delta\gamma H_p}{2}\frac{\alpha}{\beta-\alpha}\sin(\psi-\psi_H)$ so that the DW magnetization keeps on precessing. On the other hand, if $|u| \leq \left|\frac{\pi\Delta\gamma H_p}{2}\frac{\alpha}{\beta-\alpha}\right|$, $\dot{\psi} = 0$, i.e., a steady state DW motion exists, thus leading to $\dot{q} = -\frac{\beta}{\alpha}u$. In the case of $\beta = 0$, $\dot{q} = \mp\frac{\Delta}{\alpha}\dot{\psi}$ so that the DW moves only when the DW magnetization precesses, that is, the steady state does not exist. In other words, if $|u| > \left|\frac{\pi\,\Delta\gamma H_p}{2}\right|$ the DW magnetization precesses and the DW continues to move, while if $|u| \leq \left|\frac{\pi\,\Delta\gamma H_p}{2}\right|$, this corresponds to the steady state and consequently the DW stops moving. As a result, the DW velocity decreases with increasing $|H_p|$ by suppressing the DW magnetization precession, thereby forming a maximum velocity $v_{max} = |u|$ at $H_p = 0$. The DW velocity becomes zero at a threshold field, $H_{th} \equiv |H_p| = \left|\frac{2u}{\pi\,\Delta\gamma}\right|$ thus forming a dome-like $v$ versus $H_P$ curve that is symmetric with respect to $H_p = 0$. Note that $H_{th}$ depends only on $u$ and $\Delta$, thus showing that

$H_{th}$ increases with increasing $u$ and decreasing $\Delta$, which enables us to uniquely determine the values of $u$ and $\Delta$ by fitting $v_{max}$ and $H_{th}$ from $v$ versus $H_x$ curves.

Let us investigate which factors determine the width of the dome. To qualitatively understand this, let us make an aggressive approximation that $\sin\psi \sim \psi$ for $0 \le \psi \le 1$ when $\beta = 0$, $H_k = 0$, $H_p = H_x$ and $\psi_H = 0$. Then from eq (6b) and (6a), for $0 \le \psi \le 1$ we obtain $\psi \sim \frac{2u}{\gamma\pi H_x \Delta}\left[1 - \exp\left(-\frac{\pi\alpha\gamma H_x}{2(1+\alpha^2)}t\right)\right]$ and $q \sim \frac{2u}{\gamma\pi H_x \alpha}\left[1 - \exp\left(-\frac{\pi\alpha\gamma H_x}{2(1+\alpha^2)}t\right)\right]$ respectively. Hence, the time needed for the DW magnetization to reach $\psi = 1$ is $\delta t \sim -\frac{1+\alpha^2}{\alpha}\frac{2}{\pi\gamma H_x}\log\left(1 - \frac{\pi\gamma H_x \Delta}{2u}\right)$ so that the average DW velocity $v$ for $0 \le \psi \le 1$ would be $\propto \sim \frac{\alpha}{1+\alpha^2}\frac{\pi\gamma H_x}{2\log\left(1-\frac{\pi\gamma H_x\Delta}{2u}\right)}$. This shows that the dome width decreases with decreasing $\alpha$ and $H_x$ and increasing $\Delta$. As a result, the value of $\alpha$ can be uniquely determined as well.

When a longitudinal field $H_p = H_x$, i.e., $\psi_H = 0$, is applied in the presence of $H_{DM}$ and a very small $H_k$, the dome-like curve is shifted by $-H_{DM}$ thus forming a maximum at $H_x = -H_{DM}$ since $(1 + \alpha^2)\dot{\psi} = \pm\frac{\alpha}{\Delta}u - \frac{\alpha\pi\gamma(H_x+H_{DM})}{2}\sin\psi$, which is observed in our experiments (see Fig. 4 in main text). Note that when $\beta \neq 0$, the DW velocity is finite for all $H_p$ values that is different from what we observed from our experimental $v$ vs $H_x$ plot. Hence, we conclude that the non-adiabatic STT contribution in $Mn_3Z$ is very small.

Let us intuitively understand current-induced DW motion in Heusler alloys (see Supplementary Fig. 22-24). First, we assume that $H_k = H_{SH} = 0$ and $H_p = H_x < 0$ or $H_{DM} < 0$ in $\odot$ | $\otimes$ DW configuration and $|H_x|$ or $|H_{DM}|$ are large (Supplementary Fig. 21). In the absence of $J$, $H_x$ (or $H_{DM}$) stabilizes the Néel wall structure in an anti-clockwise chirality (see Supplementary Fig. 21a). When an electric current $J$ is applied along the $-\hat{x}$ direction, i.e, the conduction electrons $e$- with positive spin polarization ($P > 0$) flow along $\hat{x}$ direction, the volume spin-transfer torque $\vec{\tau}_{STT}$ starts to rotate the DW magnetization $\vec{M}$ along $+\hat{z}$ direction (Supplementary Fig. 21b). The volume STT driven motion induces a damping torque $\vec{\tau}_d = \alpha\vec{M} \times \frac{d\vec{M}}{dt}$ that rotates $\vec{M}$ along the $-\hat{\psi}$ direction, i.e. clockwise rotation from the top view (see Supplementary Fig. 21c). This leads to a non-collinearity between $\vec{M}$ and $\vec{H}_x$ (or $\vec{H}_{DM}$), thus giving rise to $\vec{\tau}_x = -\gamma\vec{M} \times \vec{H}_x$ (or $\vec{\tau}_{DM} = -\gamma\vec{M} \times \vec{H}_{DM}$). Note that $\vec{\tau}_x$ (or $\vec{\tau}_{DM}$) is always along

the $-\hat{z}$ direction, thereby compensating $\vec{\tau}_{STT}$ and slowing down the DW motion (Supplementary Fig. 21d). $\vec{\tau}_d$ rotates $\vec{M}$ along $-\hat{\psi}$ direction until $\vec{\tau}_x$ completely cancels $\vec{\tau}_{STT}$ thus stopping the DW motion at $\psi = \arcsin \frac{2u}{\pi \Delta \gamma H_x}$ (Supplementary Fig. 21e), which corresponds to $|u| \leq \left|\frac{\pi \Delta \gamma H_x}{2}\right|$, as discussed above. Here the upper bound of torque $\tau_x$ (or $\tau_{DM}$) is $\gamma M_s H_x$ (or $\gamma M_S H_{DM}$). On the other hand, if $H_x$ (or $H_{DM}$) is small, so that $\tau_{STT}$ is larger than $\gamma M_s H_x$ (or $\gamma M_S H_{DM}$), $\vec{M}$ continuously precesses and the DW keeps on moving, which corresponds to $|u| > \left|\frac{\pi \Delta \gamma H_x}{2}\right|$ (see Supplementary Fig. 22 and 25). Note that the DW velocity and precession angular velocity are maximum when the magnetization in the middle of the DW is directed along the $\hat{x}$-direction while they are minimum when the magnetization in the middle of the DW is directed along the $\hat{y}$-direction. Likewise, we can readily understand the DW motion dependence on $H_k$ (Supplementary Fig. 23) and $H_p = H_y$ (Supplementary Fig. 24). The DW width is determined by the fits to the dome in ν vs $H_x$ (Supplementary Fig. 28).

**Supplementary Note 16. Torques to move chiral domain walls and precess domain wall magnetization in wires formed from Heusler alloys**

Since out-of-plane field is not applied to perpendicularly magnetized Heusler film in our current driven DW motion experiment, Zeeman field or Zeeman-like effective induced DW motion is not taken into account. In this case, the torques that move or precess the DW are spin-transfer torque and in-plane field driven torques. First, let us consider an adiabatic STT only here. The adiabatic STT originates from total angular momentum conservation in non-uniform magnetic structure of DWs. We can readily see that adiabatic STT should be proportional to the gradient of magnetization in DW, that is, $\frac{\partial \vec{M}}{\partial x}$ when magnetic wire is in x-y plane and DW is perpendicular to x-axis. Note that the shape anisotropy of DW would favor a Bloch type wall in the absence of current. However, an effective field in x-axis caused by DMI is large consequently stabilizing a Néel DW structure and setting up a specific chirality. Note that chiral nature of DW produces effective fields (DMI fields) that have opposite direction depending on DW configurations. When a current is applied along –x direction, the spins that are polarized along +z direction exert damping-like torque $\vec{\tau}_{stt}$ that is proportional to $\frac{\partial \vec{M}}{\partial x}$ on DW magnetization that is oriented along +z direction thus rotating $\vec{M}$ toward +z direction. This motion induces the damping torque $\vec{\tau}_{stt}^{damping} = \alpha \vec{M} \times \frac{\partial \vec{M}}{\partial x}$ that rotates $\vec{M}$ clockwise direction in–plane, thus deviating $\vec{M}$ from Néel wall (see Supplementary Fig. 21). Then immediately the angle between the DW magnetization and DMI field $\vec{H}_{DMI}$ that is directed along –x direction becomes finite thus generating a torque $\vec{\tau}_{DM} = -\gamma \vec{M} \times \vec{H}_{DM}$ which is oriented along –z direction. This motion gives rise to a damping torque $\vec{\tau}_{DM}^{damping} = \alpha \vec{M} \times \frac{\partial \vec{M}}{\partial x}$ whose direction is opposite to $\vec{\tau}_{stt}^{damping}$. These all torques exactly compensate each other so that DW does not move, which corresponds to intrinsic pinning when current density is low. As the current density increases and exceed a threshold value, the adiabatic STT becomes larger than $\vec{\tau}_{DM}$ leading to a finite DW velocity. Simultaneously, $\vec{\tau}_{DM}^{damping}$ does not compensate $\vec{\tau}_{stt}^{damping}$ and DW keeps on precessing. The application of $H_x$ field on two different DW configuration break the symmetry as clearly shown from our experiment, thus revealing the chiral nature of DW in Heusler alloys. Note that, with a given

chirality, the DWs move in the same direction and same velocity under SOT in the absence of externally applied field.

Next, let us investigate the DMI+SOT. The spin accumulation that is orientated along –*y* direction induces damping-like torque (SOT) on the DW when the spin Hall angle is positive and current is flow +*x*-direction. In the same way as the STT does as shown above, this rotates the DW magnetization away from x-axis thus inducing a finite angle between DW magnetization and DMI effective. As a result, $\vec{\tau}_{DM} = -\gamma\vec{M} \times \vec{H}_{DM}$ , i.e., DMI torque, is generated along z-axis thus moving the DWs.

**Supplementary Note 17. Upper bound estimation of narrow DW width in the ultra-thin $Mn_3Z$ Heuslers**

The DW width equals $\pi\Delta$ with $\Delta$ being the DW width parameter, equal to $\sqrt{A_{ex}/K_{eff}}$ where exchange stiffness is $A_{ex}$ and effective anisotropy $K_{eff} = K - 2\pi M_s^2 = \frac{H_K M_S}{2} - 2\pi M_s^2$. Note that K, $H_K$ and $M_S$ are crystalline anisotropy constant, crystalline anisotropy field and saturation magnetization, respectively. For the case of $Mn_3Ge$, we estimate the exchange stiffness $A_{ex} \sim \frac{J_{ex}}{a} = \frac{72\ \mathrm{meV}}{5\ \text{Å}} = 2.3 \times 10^{-11}\mathrm{Joule/m}$ (cf. $A_{ex} \sim 1.9 \times 10^{-11}\ \mathrm{Joule/m}$ for Co). Our estimated magnetization $M_S \sim 110\ \mathrm{emu/cc}$ (from our measurements supported by DFT calculations) and an anisotropy field $H_{eff} > 7$ T (the limit of our Quantum Design SQUID-VSM magnetometer). Thus, we estimate, $\Delta < \sqrt{\frac{A_{ex}}{K_{eff}}} = \sqrt{\frac{2A_{ex}}{M_S H_{eff}}} = \sqrt{\frac{2\times 2.3\times 10^{-11}\quad nm^2}{110\times 7\times 10^4\times 10^{-7}/10^{-6}}} = 7.7$ nm. Please note that 7.7 nm is the upper bound for $\Delta$. We note that due to the significant magnetization from the MgO substrates and the tiny magnetization of the Heusler material, it is very difficult to precisely determine the thickness of any dead layer but it is clear from the magnetic measurements that this is less than a fraction of a unit cell equivalent thickness. On the other hand, we can measure the anisotropy of the magnetization reliably and the Heusler films show very high anisotropy values in unit cell thick Heusler layers – so large that we cannot rotate their magnetization in plane using available magnetic fields (7T) in a commercial SQUID magnetometer. This is why our paper focuses predominantly on PMA which we show we have been able to produce in single unit cell thick Heusler films for the very first time.

**Supplementary Note 18. One-dimensional model simulation of $\nu - J$ curves**

We reproduce $\nu - J$ curve for $Mn_3Sb$ (Fig. 3e) with 1D analytical model as shown in Supplementary Fig. 29. Although non-adiabatic spin-transfer-torque parameter $\beta$ is set to be zero in our simulation, the intrinsic pinning is small since $H_k$ and $H_{\mathrm{DM}}$ are small. In addition, a finite SOT gives rise to $J_c = 0$ which is apparently far from what we observe from the experiment. Hence, a finite $J_{\mathrm{c}}$ is due to the extrinsic pinning that can be induced by inhomogeneous anisotropy or other reasons. Here to emulate the extrinsic pinning, periodic pinning potential is used (see the details in ref. 5 and 8). Note that the fitted pinning potential for 10 Å $Mn_3Sb$ is significantly smaller than that for 20 Å $Mn_3Sb$. That could infer that $Mn_3Sb$ becomes rougher with the increasing thickness thus increasing the anisotropy inhomogeneity.

**Supplementary Note 19. Comparison of bulk DMI constant to Heisenberg exchange interaction constant**

Let us consider the case of $Mn_3Sn$. The bulk DMI constant $D = \frac{H_{DM}M_S}{2} \cong 1.2 \times 10^5$ erg/cm$^3$, as derived from $H_{\mathrm{DM}} = 1$ kOe and $M_s$=250 emu/cm$^3$. The calculated Heisenberg exchange interaction constant $J_{ex} = 33$ meV/(5×10$^{-8}$ cm)$^3$ ≅ 4.2×10$^9$ erg/cm$^3$ based on the unit cell volume ~ (5×10$^{-8}$ cm)$^3$ [1]. Consequently, we obtain the ratio, $\frac{D}{J_{ex}} \cong 2.9 \times 10^{-5}$, and the canting angle of the moments from a collinear alignment in the domain is, $\eta \cong \frac{1}{2}\tan^{-1}\frac{D}{J_{ex}} = 0.009°$ [2].

| | 7.5 Å $Mn_3Sn$ | 7.5 Å $Mn_3Sn$ (CoGa capped) | 10 Å $Mn_3Sn$ | 10 Å $Mn_3Sn$ (CoGa capped) | 15 Å $Mn_3Sn$ | 20 Å $Mn_3Ge$ | 20 Å $Mn_3Sb$ | 20 Å $Mn_2Sb$ on CoSn | 20 Å $Mn_{2.7}Sb$ on CoAl |
|---|---|---|---|---|---|---|---|---|---|
| $\alpha$ | 0.048 | 0.048 | 0.025 | 0.03 | 0.02 | 0.01 | 0.05 | 0.05 | 0.09 |
| $\beta$ | 0 | 0 | 0 | 0 | 0 | 0 | 0 | 0 | 0 |
| Δ (Å) | 5 | 1.15 | 5 | 0.7 | 8 | 0.3 | 12 | 12 | 14 |
| $H_k$ (Oe) | 100 | 100 | 100 | 100 | 100 | 100 | 100 | 100 | 100 |
| $H_{SH}$ (Oe) | 100 | 0 | 13 | 0 | 5 | 5 | 10 | 20 | 5 |
| $\theta_{SH}$ | 0.0114 | 0.0000 | 0.0012 | 0.0000 | 0.0006 | 0.0012 | 0.0049 | 0.0055 | 0.0013 |
| $u$ (m/s) | 15 | 17 | 30 | 4 | 45 | 4 | -27 | -15 | -24 |
| $H_{DM}$ (Oe) | -1000 (CCW) | -1000 (CCW) | -1000 (CCW) | -1000 (CCW) | -1000 (CCW) | 1500 (CW) | 300 (CW) | 50 (CW) | 350 (CW) |

**Supplementary Table 2**. List of fitting parameters for the fits in Fig. 4 and Fig. 5a-d of the main text and fits to data in Supplementary Fig. 11-13. The corresponding fits of Supplementary Fig. 13, are plotted in Supplementary Fig. 26-27.

| | $Mn_{3.3}Sb$ on CoGa | $Mn_3Sb$ on CoGa | $Mn_{2.5}Sb$ on CoGa | $Mn_{2.4}Sb$ on CoGa | $Mn_2Sb$ on CoGa | $Mn_3Sb$ under 5 Pt | $Mn_3Sb$ under 10 Pt | $Mn_3Sb$ under 20 W |
|---|---|---|---|---|---|---|---|---|
| $\alpha$ | 0.05 | 0.05 | 0.07 | 0.05 | 0.05 | 0.03 | 0.02 | 0.12 |
| $\beta$ | 0 | 0 | 0 | 0 | 0 | 0 | 0 | 0 |
| $\Delta$ (Å) | 12 | 14 | 12 | 14 | 14 | 8 | 2.5 | 30 |
| $H_k\ (Oe)$ | 100 | 100 | 100 | 100 | 100 | 100 | 100 | 100 |
| $H_{SH}\ (Oe)$ | 10 | 10 | 10 | 10 | 10 | 7 | -3 | 200 |
| $\theta_{SH}$ | 0.0024 | 0.0048 | 0.0046 | 0.0047 | 0.0050 | 0.0013 | -0.0006 | 0.0838 |
| $u\ (m/s)$ | -39 | -25.5 | -11 | -12 | -9 | -23 | -14 | -10 |
| $H_{DM}(Oe)$ | 700 (CW) | 350 (CW) | 200 (CW) | 150 (CW) | 50 (CW) | 150 (CW) | 800 (CW) | 450 (CW) |

**Supplementary Table 3**. List of parameters to fit plots in Fig. 5e-f of main text. Also for the fitted curves in Supplementary Fig. 15-18.

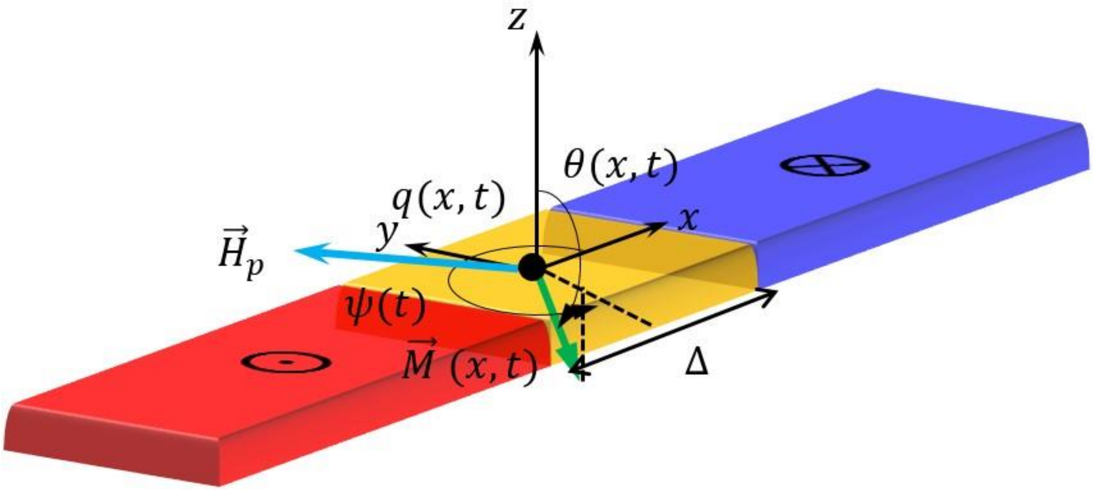


**Supplementary Figure 20**. Schematic of parameters used in 1D model calculation for ⊙ | ⊗ DW configuration.

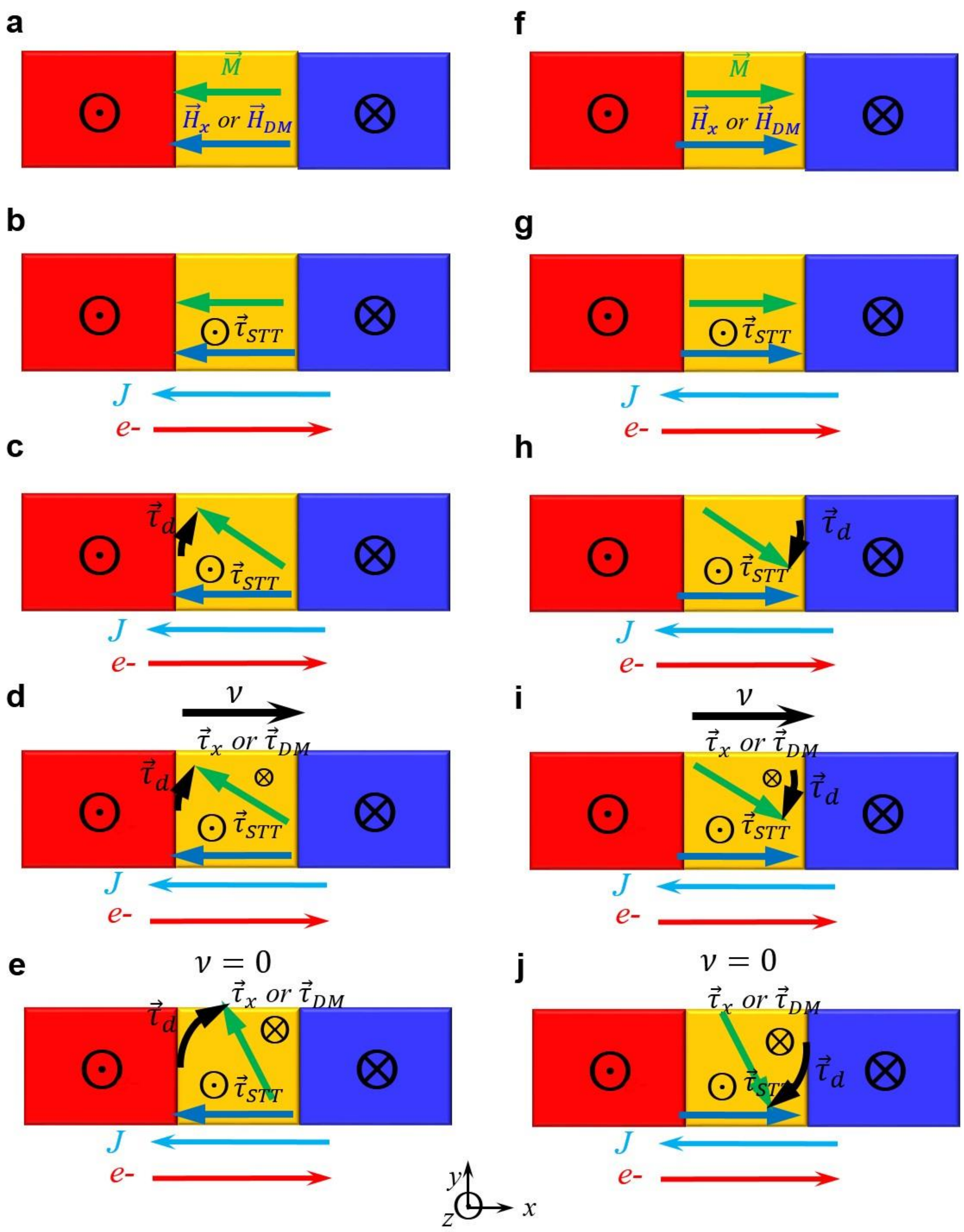


**Supplementary Figure 21**. Top view cartoon of current driven DW motion with ⊙ | ⊗ DW configuration in the presence of $H_x$ or $H_{DM}$ only. The torque $\vec{\tau}_x$ or $\vec{\tau}_{DM}$ generated by these fields are larger than $\vec{\tau}_{STT}$ here. $P > 0$ is considered. **(a)**, Initially $J = 0$. **(b)-(j)**, $J < 0$.

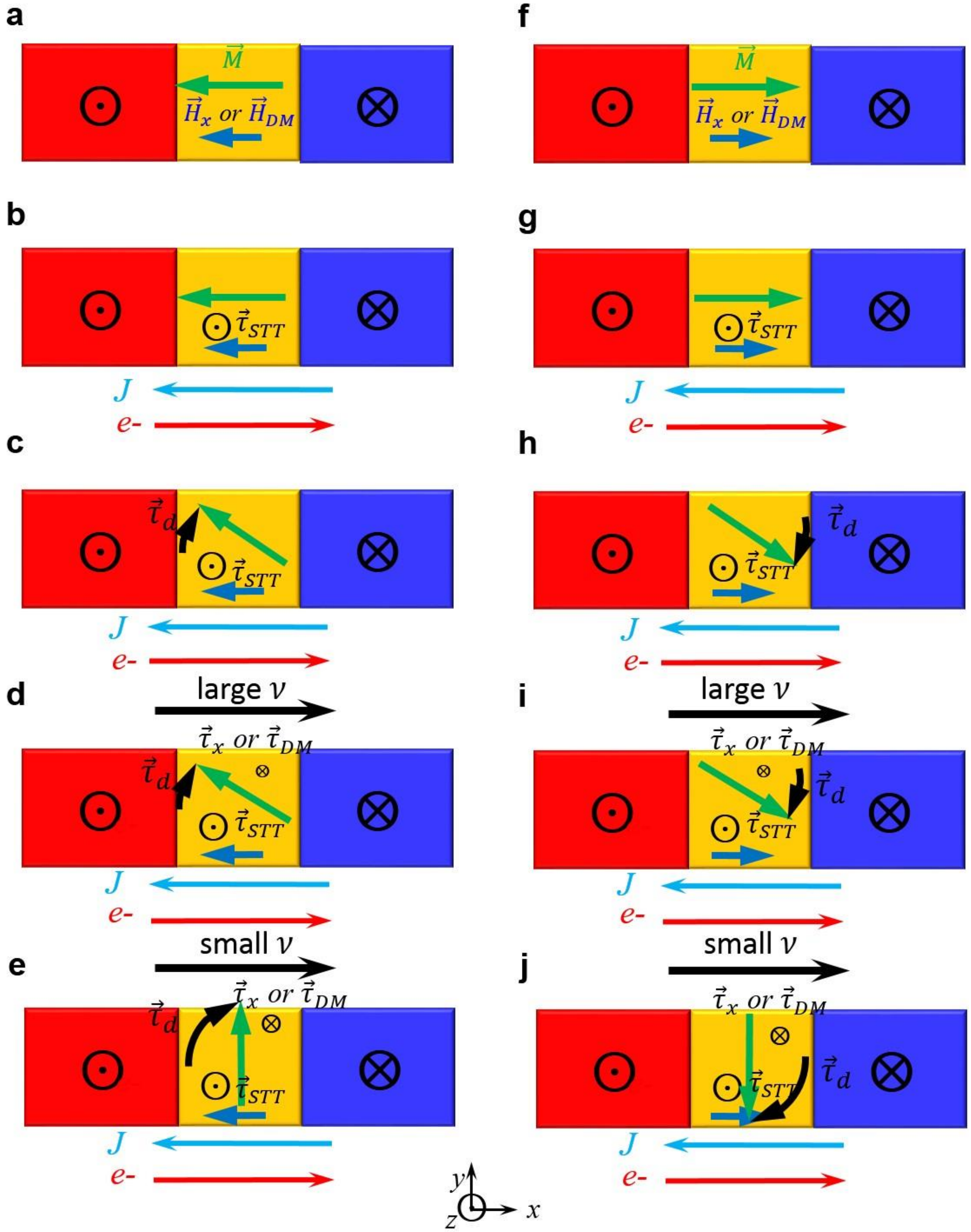


**Supplementary Figure 22**. Top view cartoon of current driven DW motion with ⊙ | ⊗ DW configuration in the presence of $H_x$ or $H_{DM}$ only. The torque $\vec{\tau}_x$ or $\vec{\tau}_{DM}$ generated by these fields are smaller than $\vec{\tau}_{STT}$ here. $P > 0$ is considered. **(a)**, Initially $J = 0$. **(b)-(d)**, $J < 0$.

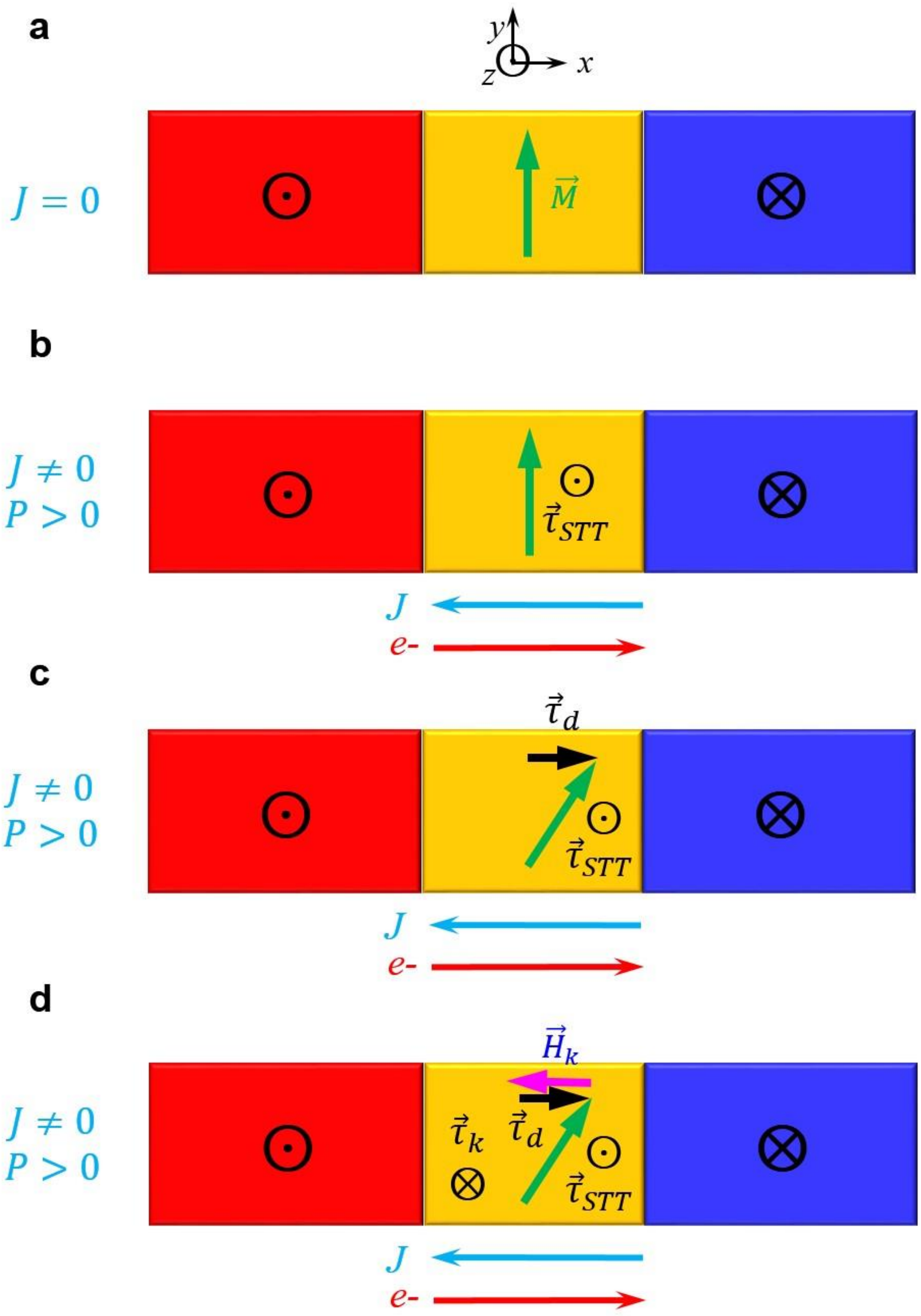


**Supplementary Figure 23**. Top view cartoon of current driven DW motion with ⊙ | ⊗ DW configuration in the presence of $H_k$ only. **(a)**, Initially $J = 0$. **(b)-(d)**, $J < 0$.

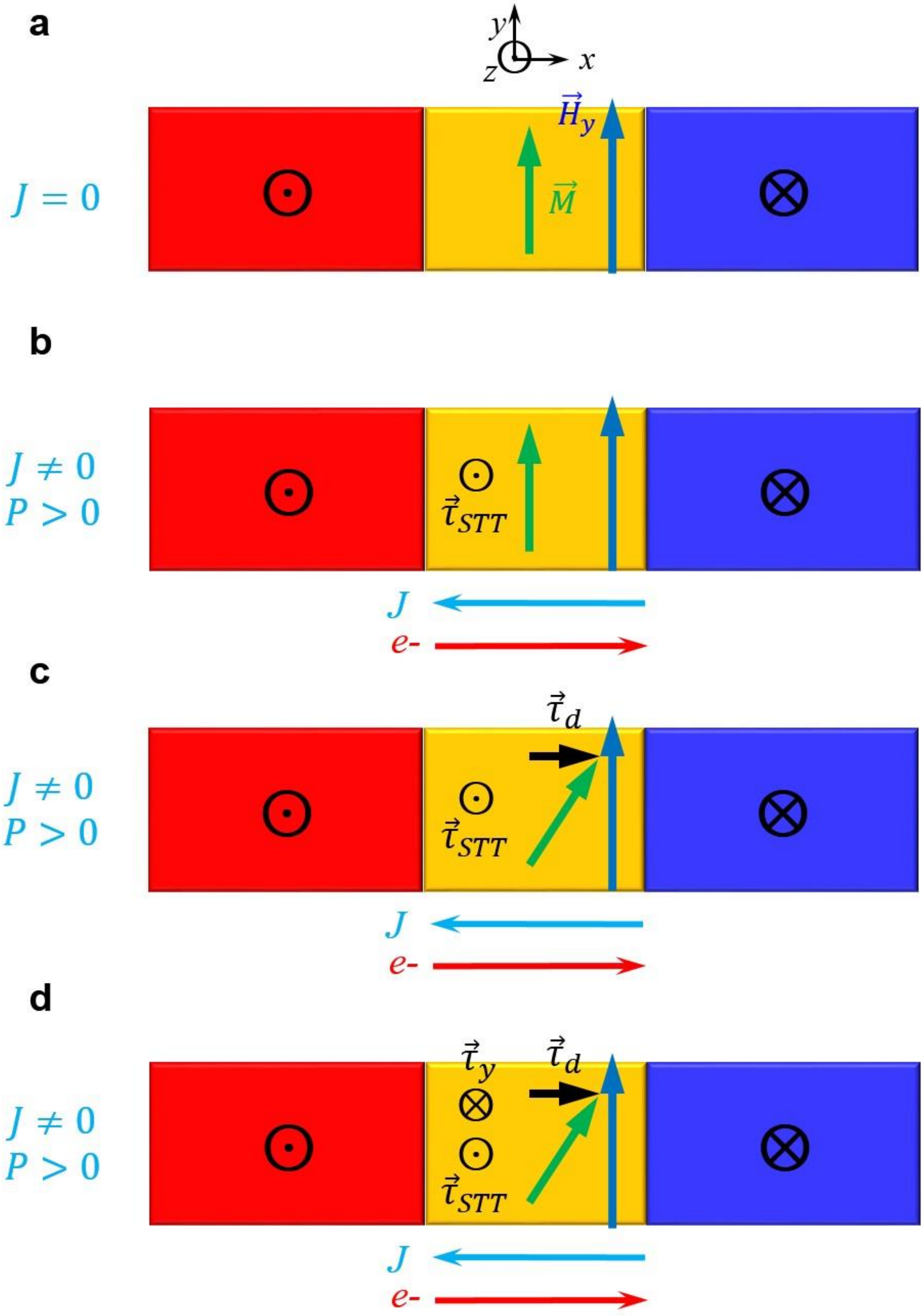


**Supplementary Figure 24**. Top view cartoon of current driven DW motion with ⊙ | ⊗ DW configuration in the presence of $H_y$ only. **(a)**, Initially $J = 0$. **(b)-(d)**, $J < 0$.

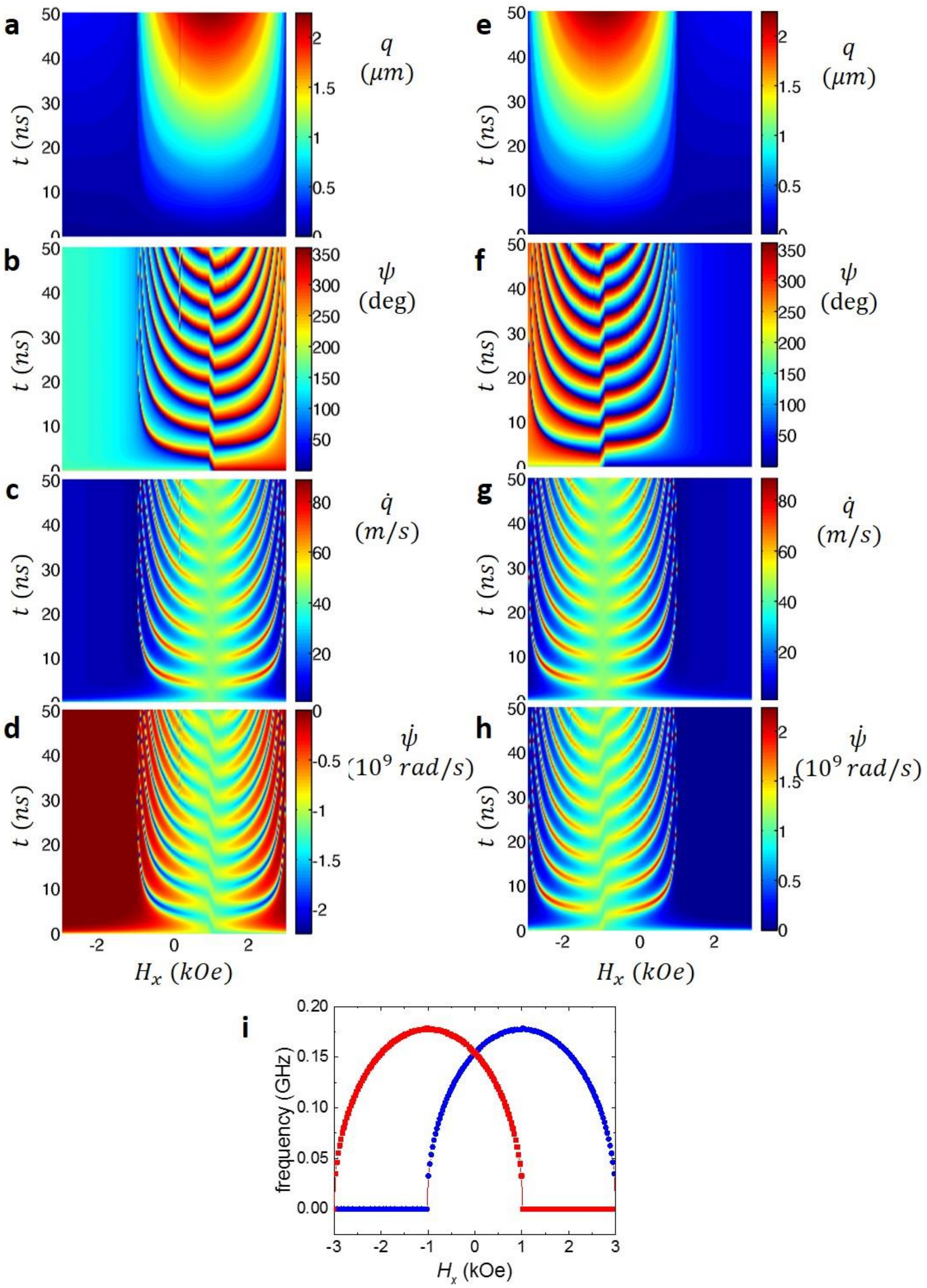


**Supplementary Figure 25**. **(a)-(h),** 2D contour plots based on the 1D model of time resolved **(a),(c)**, $q$, **(b),(f)**, $\psi$, **(c),(g)**, $\dot{q}$, and **(d),(h)**, $\dot{\psi}$ versus $H_x$ for ⊙ | ⊗ **(a)-(d)** and ⊗ | ⊙ **(e)-(h)**. **(i)**, Plot of precession frequency of DW magnetization versus $H_x$. The red and blue line and symbols correspond to ⊙ | ⊗ and ⊗ | ⊙ configurations, respectively. The parameters used in the calculation are $\alpha = 0.025$, $\beta = 0$, $\Delta$= 0.5 nm, $H_k$ = 100 Oe, $H_{\mathrm{SH}}$ = 0 Oe, $u$ = 30 m/s, and $H_{\mathrm{DM}} = -1$ kOe (⊙ | ⊗) and 1 kOe (⊗ | ⊙).

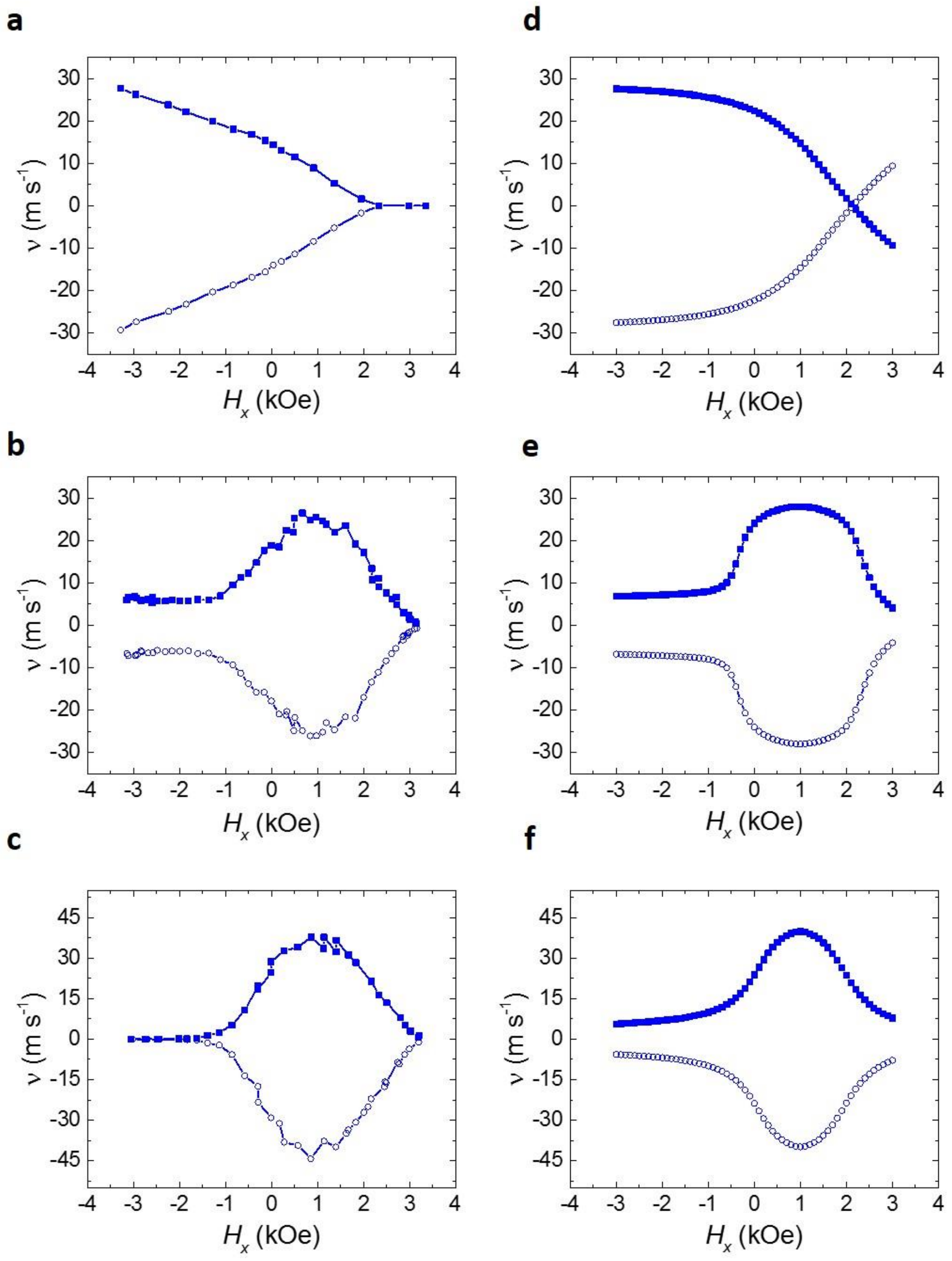


**Supplementary Figure 26**. Plots of ⊙ | ⊗ DW velocity versus $H_x$ measured from 2 μm wide and $x$ nm thick $Mn_3Sn$ nanowires **(a)-(c)** and corresponding 1D model calculations **(d)-(f)** where $x = 7.5$ **(a),(d)**, 10 **(b),(e)**, and 15 **(c),(f)**, all in Å. Solid and open symbols correspond to $J > 0$ and $J < 0$, respectively. The fitted parameters are summarized in Supplementary Table 2.

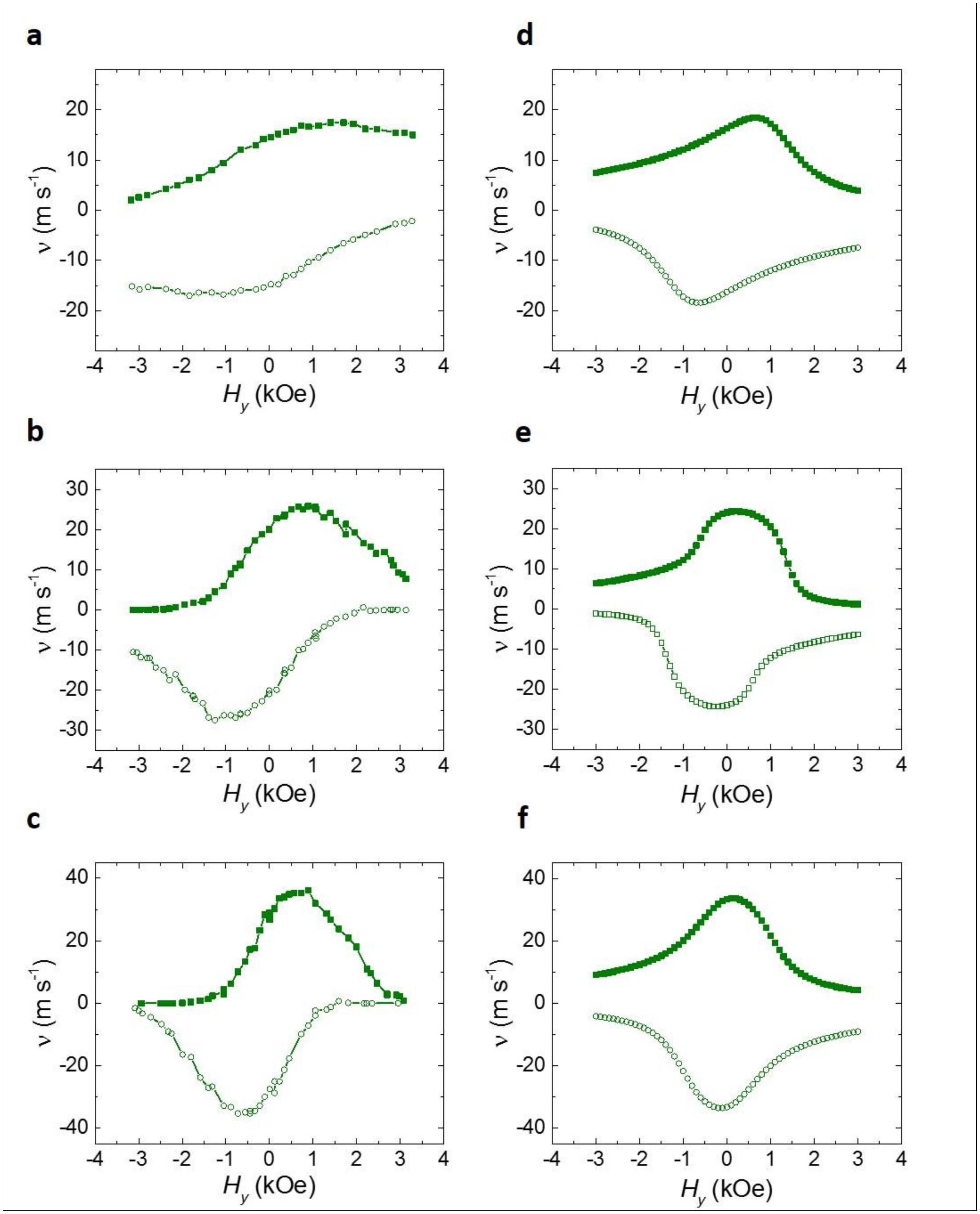


**Supplementary Figure 27**. Plots of ⊙ | ⊗ DW velocity versus $H_y$ measured from 2 μm wide and $x$ nm thick $Mn_3Sn$ nanowires **(a)-(c)** and corresponding 1D model calculations **(d)-(f)** where $x = 7.5$ **(a),(d)**, 10 **(b),(e)**, and 15 **(c),(f)**, all in Å. Solid and open symbols correspond to $J > 0$ and $J < 0$, respectively. The fitted parameters are summarized in Supplementary Table 2.

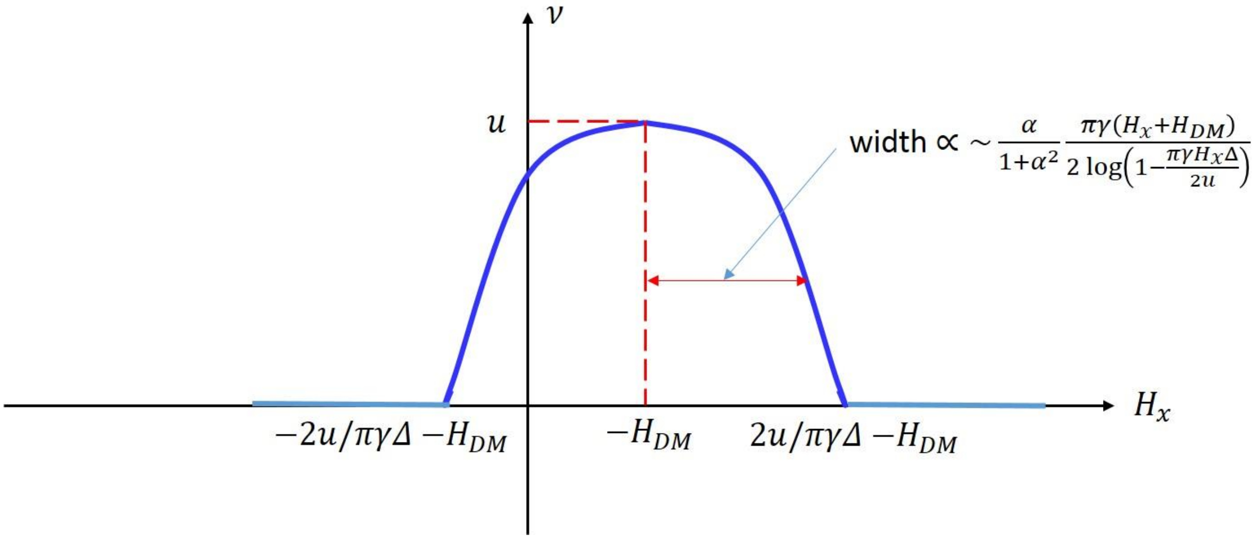


**Supplementary Figure 28**. 1D model approximation of the DW width Δ, fitting the DW velocity versus $H_x$ applied field.

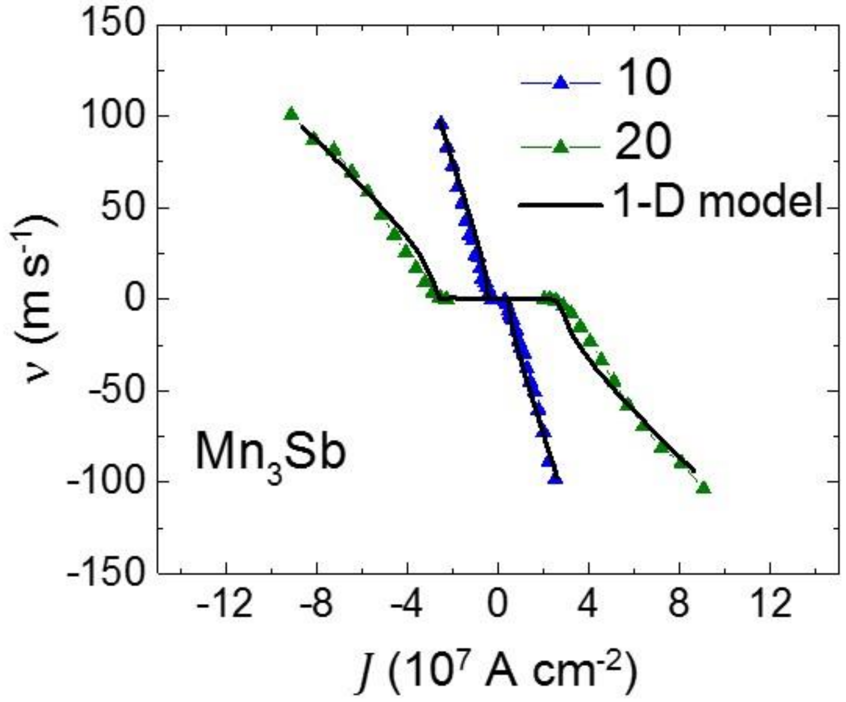


**Supplementary Figure 29**. DW velocity versus current density $J$ for experimental results shown in Fig. 3e. Symbols and thick lines correspond to experiment and model, respectively. The commonly fitted parameters for two curves are $\Delta = 1.2\ nm, H_k = 100\ Oe$, and $H_{\mathrm{DM}} = 50\ Oe$. 10 Å $Mn_3Sb$: $\alpha = 0.07$, $M_{\mathrm{S}} = 60\ emu/cc\ \ P = 0.4$, and $V_{\mathrm{pinning}} = 1.5 \times 10^4$. 20 Å $Mn_3Sb$: $\alpha = 0.05$, $M_{\mathrm{S}} = 100\ \mathrm{emu/cc}$, $P = 0.2$, and $V_{\mathrm{pinning}} = 5 \times 10^4$.

**Supplementary References**

[1] Faleev, S. V. et al. *Origin of the Tetragonal Ground State of Heusler Compounds,* Phys. Rev. Applied **7,** 034022 (2017).

[2] A. P. Malozemoff, & J. C. Slonczewski, *Magnetic Domain Walls in Bubble Materials*, (Academic, New York, 1979).

[3] A. Thiaville, Y. Nakatani, J. Miltat, & Y. Suzuki, *Micromagnetic understanding of current-driven domain wall motion in patterned nanowires*. Europhys. Lett. 69, 990-996 (2005).

[4] Kwang-Su Ryu, Luc Thomas, See-Hun Yang, & Stuart S.P. Parkin, *Chiral Spin Torque at Magnetic Domain Walls,* Nature Nanotechnology **8**, 527–533 (2013).

[5] Kwang-Su Ryu, See-Hun Yang, Luc Thomas, & Stuart S.P. Parkin, *Chiral Spin Torque Arising from Proximity Induced Magnetization*. Nature Communications **5**, 3910 (2014).

[6] See-Hun Yang, Kwang-Su Ryu, & Stuart S.P. Parkin, *Domain-wall velocities of up to 750 m s−1 driven by exchange-coupling torque in synthetic antiferromagnets*, Nature Nanotechnology **10**, 221 (2015).

[7] A. Hubert, *Theorie der Domänenwände in Geordneten Medien* (Springer, Berlin, 1974).

[8] Chirag Garg, See-Hun Yang, Timothy Phung, Aakash Pushp, & Stuart S.P. Parkin, *Dramatic influence of curvature of nanowire on chiral domain wall velocity*. Science Advances **3**, e1602804 (2017).
